# Characterising the association between posterior parietal metabolite levels and cortical macrostructure in a cohort spanning childhood to adulthood

**DOI:** 10.1162/IMAG.a.1041

**Published:** 2025-12-03

**Authors:** Alice R. Thomson, Duanghathai Pasanta, Richard A.E. Edden, Tomoki Arichi, Xiaoqian Chai, Nicolaas A. Puts

**Affiliations:** Department of Forensic and Neurodevelopmental Sciences, Institute of Psychiatry, Psychology, and Neuroscience, King’s College London, London, United Kingdom; MRC Centre for Neurodevelopmental Disorders, King’s College London, London, United Kingdom; Department of Radiologic Technology, Faculty of Associated Medical Sciences, Chiang Mai University, Chiang Mai, Thailand; Russell H. Morgan Department of Radiology and Radiological Science, The Johns Hopkins University School of Medicine, Baltimore, MD, United States; F.M. Kirby Research Centre for Functional Brain Imaging, Kennedy Krieger Institute, Baltimore, MD, United States; Research Department of Early Life Imaging, School Biomedical Engineering and Imaging Sciences, King’s College London, London, United Kingdom; Department of Neurology and Neurosurgery, McGill University, Montreal, QC, Canada

**Keywords:** macrostructure, GABA, glutamate, Glx, development, childhood, parietal cortex

## Abstract

Postnatal brain development is characterised by dynamic macrostructural changes, including cortical thinning and cortical flattening during childhood and adolescence. These macrostructural changes are parallel with developmental changes in brain neurochemistry, probed in the human brain using magnetic resonance spectroscopy (MRS). This includes neurotransmitters such as glutamate and gamma-aminobutyric acid (GABA), as well as building blocks of neuronal and associated tissue such as N-acetyl aspartate (NAA), and those involved in metabolism such as creatine (Cr). While previous research has linked MRS-measured neuro-metabolite levels to bulk tissue composition (e.g., grey matter, white matter, and cerebrospinal fluid), the relationship between neurochemistry and more granular macrostructural metrics, such as cortical thickness, area, volume, and local gyrification, remains unexplored. This study investigates the association between MRS-measured neuro-metabolite levels in the posterior parietal cortex (PPC) and PPC-voxel cortical macrostructural metrics in a developmental cohort of 86 individuals aged 5–35 years. Our findings reveal significant and positive age-dependent associations between PPC cortical thickness, volume, local gyrification index (LGI), and Glx (glutamate + glutamine) levels, likely because differences in cortical *microstructure*, including dendritic arbour complexity, contributes to variation in both local cortical macrostructure and Glx activity across development. We further show age-independent associations between PPC Cr and local cortical surface area and volume, suggesting that inter-individual variability in these cortical macrostructural metrics is linked to underlying tissue energetic properties. These results highlight the importance of accounting for macrostructural characteristics when interpreting the neuroanatomical correlates of MRS-measured neuro-metabolites, beyond controlling for bulk tissue composition. This approach is particularly crucial when comparing neuro-metabolite levels across groups with known structural differences, such as developmental cohorts or individuals with neurodevelopmental conditions.

## 1 Introduction

Postnatal brain development is characterised by dynamic changes in brain macrostructure from childhood to adulthood, including cortical thinning (Baik et al., 2023; Ducharme et al., 2016; Frangou et al., 2022; Koolschijn & Crone, 2013; Muftuler et al., 2011) and decreases in cortical folding (Alemán-Gómez et al., 2013; Ducharme et al., 2016; Klein et al., 2014; White et al., 2010). Cortical thinning during childhood and adolescence is thought to reflect the developmental remodelling of laminar structural complexity driven by dendritic arbour pruning (la Fougère et al., 2011; Petanjek et al., 2011; Sowell et al., 2004; Vidal-Pineiro et al., 2020). Synaptic connections in the cortex are initially formed in excess, peaking in early childhood (2–4 years of age; Huttenlocher, 1979), before weaker connections are subsequently eliminated from childhood to early adulthood (Faust et al., 2021; Huttenlocher, 1979; Huttenlocher & Dabholkar, 1997; Huttenlocher & de Courten, 1987; Selemon, 2013). This achieves highly efficient, adaptive, and precise connectivity, as the fewer remaining synapses are maintained and strengthened (Bourgeois et al., 1989, 1994; Geschwind, 2011; Huttenlocher & Dabholkar, 1997; Huttenlocher & de Courten, 1987; Petanjek et al., 2011; Selemon, 2013; Zecevic et al., 1989). This process of structural refinement in the cortex is closely linked to cognitive development (Fleming & McDermott, 2024). Similarly, gyrification or cortical folding is a developmental process whereby the cortical surface is folded, increasing cortical surface area within a fixed volume to support efficient structural connectivity (White et al., 2010), and making functionally distinct brain areas spatially isolated (Jiang et al., 2021). While cortical folding predominantly occurs during the prenatal and early postnatal period (White et al., 2010), gradual decreases in local gyrification index (LGI), a measure of cortical folding (lower LGI indicating a lesser degree of cortical folding), are observed across childhood and adolescence (Alemán-Gómez et al., 2013; White et al., 2010). This has been linked to changes in dendritic morphology and synaptic connectivity also, which alters cortical structural connectivity and so folding forces, leading to decreased sulcal depth and a gradual flattening of the cortex (Alemán-Gómez et al., 2013; White et al., 2010).

Such developmental changes in brain macro-architecture are concurrent with developmental changes in brain neurochemistry, probed *in vivo* using magnetic resonance spectroscopy (MRS). This includes neurotransmitters such as gamma-aminobutyric acid (GABA) and glutamate, as well as building blocks of neuronal and associated tissue such as N-acetyl aspartate (NAA), choline (Cho), and those involved in metabolism such as creatine (Cr) and myo-inositol (mI; Rae, 2014). Recent studies suggest that MRS neuro-metabolites undergo developmental shifts that parallel changes in cortical macrostructure across childhood and adolescence (Mikkelsen et al., 2017; Perdue et al., 2023; Reyngoudt et al., 2012; Thomson, Hwa, et al., 2024), while strong associations between cortical thickness and genes related to neuronal metabolism and function further support a potential link between these macrostructural and neurochemical changes (Zhou et al., 2023).

Despite these findings, the association between changes in neurochemistry and finer-grained structural metrics, such as cortical thickness, area, volume, and cortical folding, has yet to be explored in a single neurotypical cohort. Prior studies have primarily established links between neuro-metabolite concentrations and bulk voxel tissue composition (gross grey matter (GM), white matter (WM), and cerebral spinal fluid (CSF)), limiting the ability to draw detailed conclusions about the structural basis of neuro-metabolite differences. Macrostructural metrics, such as cortical thickness and LGI, provide more detailed insights into cortical architecture, and developmental changes in these measures are likely to interact with underlying biochemical processes, shaping regional brain development.

To address this gap, this study is the first to investigate the associations between MRS-measured neuro-metabolite levels in the posterior parietal cortex (PPC), which we previously characterised (Thomson, Hwa, et al., 2024), and macro-structural metrics derived from the same PPC MRS voxel in a developmental cohort spanning 5 to 35 years of age. The PPC voxel was selected due to its more protracted development (Hill et al., 2010; Kaas & Stepniewska, 2016), enabling us to associate developmental changes in macrostructure and neurochemistry from mid-childhood through early adulthood. Understanding the neuroanatomical correlates of changes in regional brain chemistry across this period is valuable for a more detailed characterisation of typical brain development. It also offers a more comprehensive framework for understanding the neurobiological underpinnings of neurodevelopmental disorders, in which both neurochemistry *and* cortical macrostructure have been shown to be disrupted, and may be linked (Ecker et al., 2016; Hyde et al., 2010; Kohli et al., 2019; Puts, Wodka, et al., 2017; Thomson, Pasanta, et al., 2024).

## 2 Methods

### 2.1 Participants

One hundred and seventeen participants underwent *in vivo* magnetic resonance imaging (MRI), overseen through local Kennedy Krieger Institute and Johns Hopkins University Institutional Review Board (IRB) procedures. Children assented, and their parents consented, to participation. All participants were native English speakers, right-handed, had normal or corrected-to-normal vision, with no history of psychiatric, neurological, or developmental disorders. All participants had IQ > 85. Participant demographic data were collected at the time of scan. Eighty-six of these participants, with high-quality MRS and structural MRI data, were included in this analysis, aged between 5 and 35 years of age (Table 1). Data quality was assessed both across and within age groups (children, adolescents, and adults) to examine potential quality bias associated with age (Table 1 & Supplementary Table 1).

**Table 1.** Sample demographics by age group.

	N (females/males)	median age (IQR)	median IQ (IQR)
Child (5 ≥ age < 12)	31 (15/16)	10.135 (2.58)	115 (17.25)
Adolescent (12 ≥ age < 18)	22 (10/12)	14.41 (2.25)	113 (15.00)
Adult (age ≥ 18)	33 (15/18)	21.67 (7.67)	120.00 (10.00)
All participants	86 (40/46)	14.71 (9.52)	117.00 (16.25)

Note only participants with high-quality MRS and structural MRI data were included.

IQR = interquartile range.

### 2.2 MRI

#### 2.2.1 Structural data

MRI was performed on a Philips 3 Tesla Achieva scanner at the F.M. Kirby Research Centre for Functional Brain Imaging at the Kennedy Krieger Institute in Baltimore, USA, with a 32-channel receive head coil. High-resolution (1 mm^3^ isotropic) T_1_-weighted magnetisation-prepared rapid acquisition gradient-echo sequence (MPRAGE) anatomical images were acquired (slice thickness: 0.83 mm; in-plane resolution: 1 × 0.83 mm; TR: 7 ms; TE: 3.2 ms), reconstructed, and visually inspected for artefacts (by AT).

#### 2.2.2 MRS data

MRS data reported here have been reported previously in Thomson, Hwa, et al. (2024) in accordance with reporting standards (Lin et al., 2021)—see Supplementary Table 1. MRS data were acquired from a 27-ml voxel (3 cm × 3 cm × 3 cm) placed over the posterior parietal cortex (PPC) and centred on the midline (Fig. 2). MRS was performed using GABA-selective MEshcher-Garwood Point RESolved Spectroscopy (MEGA-PRESS; (Mescher et al., 1998)); 320 transients (160 ON and 160 OFF), 2048 data points, TE/TR 68/2000 ms, with editing pulses placed at 1.9 ppm in the edit-ON acquisitions and 7.46 ppm in the edit-OFF acquisitions, and using VAPOR water suppression. An interleaved unsuppressed water reference with the same parameters as water suppressed scans (but with only 16 averages) was used to mitigate scanner drift and for subsequent eddy current and phase corrections, and for metabolite quantification (Edden et al., 2016; Mikkelsen, Tapper, et al., 2020). Thirty-one MRS datasets were excluded due to poor data quality (for details see Thomson, Hwa, et al., 2024). As such, our MRS and structural data analysis use data from 86 participants. Demographics for this cohort are shown in Table 1.

### 2.3 Structural data processing

T_1_-weighted images were motion corrected, transformed, intensity corrected, and segmented in FreeSurfer (version 7.4.1), which shows reliable accuracy across age groups, including 5-year-old children (Pulli et al., 2022). Images were segmented (into GM, WM, CSF volumes) and the cortical surface was reconstructed (Dale et al., 1999; Fischl & Dale, 2000; Fischl et al., 1999). Surface reconstructions were inspected for accuracy per participant and adjusted where necessary (< 1% of data). The FreeSurfer commands *recon-all* and *mris_compute_lgi* were used to calculate local gyrification index (LGI) for each point on the cortical surface (typically scoring between 1 and 5); first, a smooth outer surface mask, enveloping the pial surface, is created for each participant. The smooth outer surface mask and the pial surface are segmented into smaller, identical circular regions from which the local gyrification is calculated for each vertex, this being the ratio of the area of the pial surface at the outer smooth mask to the area of the smooth outer surface mask (Schaer et al., 2008). Estimated intracranial volume (eTIV, in mm^3^) was also extracted from the results of the FreeSurfer procedures for each participant.

#### 2.3.1 Extracting voxel surface metrics

**Fig. 1.**
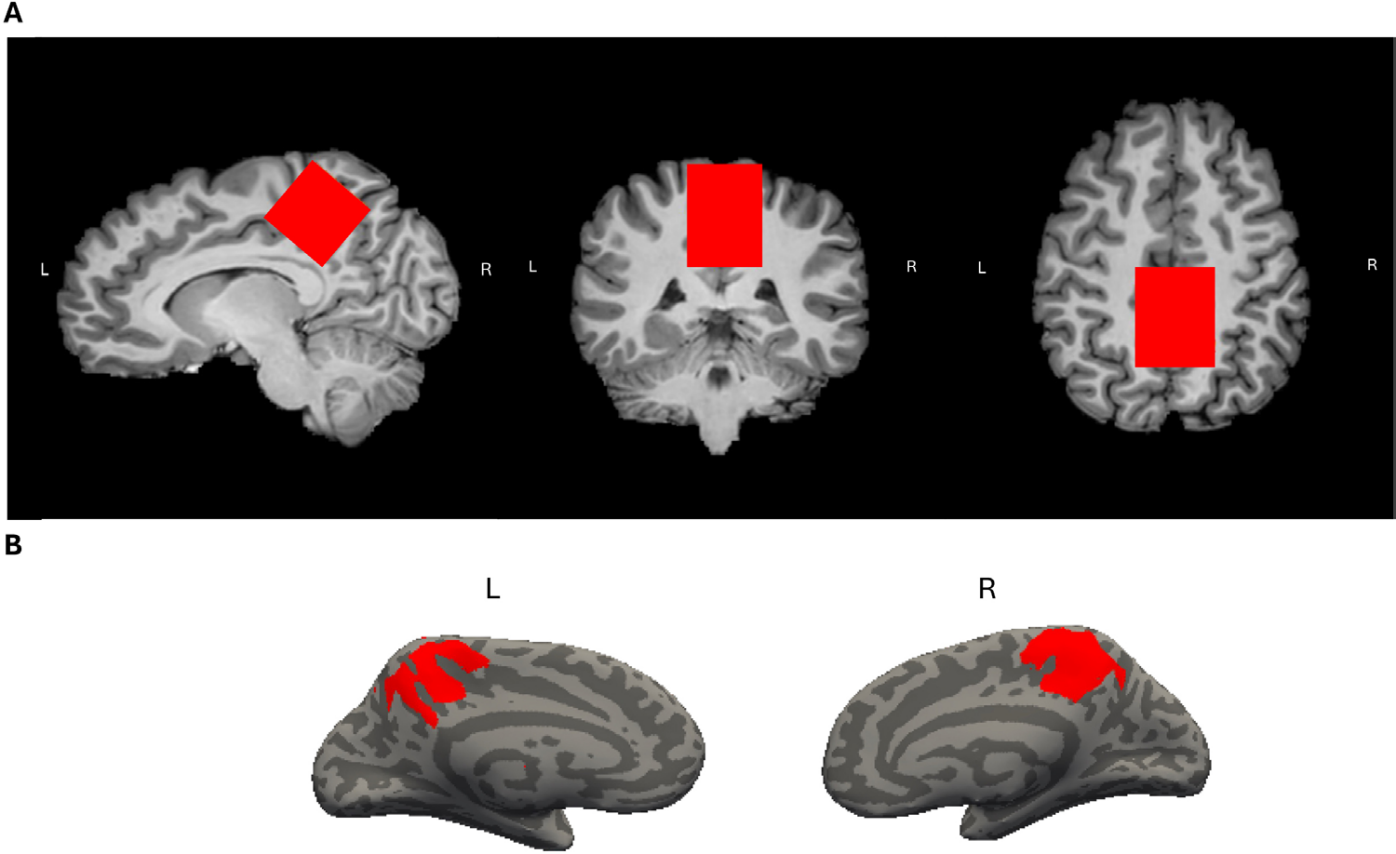
(A) Example PPC voxel mask mapped onto the corresponding T_1_-weighted image. (B) The same PPC voxel mask projected onto an inflated cortical surface of the left and right hemispheres (cortical surfaces were obtained using FreeSurfer).

The Freesurfer command *mri_vol2surf* was used to project the PPC MRS voxel mask onto the cortical surface (pial). This was performed individually for each participant as voxel placement varied based on individual brain anatomy. As the MRS PPC voxel was centred to the midline, the voxel volume was projected into the medial parts of both the right and left hemispheres, creating two voxel masks (see Fig. 1). The Freesurfer *MRI_segstats* command was then used to extract quantitative measures from the 2D voxel masks projected onto the inflated right and left hemisphere cortical surface. Measures extracted included mean cortical thickness, which is the shortest distance between the pial and white matter surface (mm), cortical volume (mm^3^), and mean voxel LGI (Schaer et al., 2008). Per PPC voxel structural metric, mean values were calculated from right and light hemisphere estimates and used for all analyses described below.

### 2.4 MRS data processing

Raw MRS data were processed using Osprey (Version 2.4.0; Oeltzschner et al., 2020; version 2022a). First, raw data were eddy current corrected using the water reference followed by frequency and phase correction using robust spectral registration (Mikkelsen, He, et al., 2020; Near et al., 2011; Oeltzschner et al., 2020), Fourier transformation, and water signal removal. Edit-ON and edit-OFF spectra averages were subtracted to resolve GABA+ (at 3.02 ppm) in the difference spectrum (GABA-DIFF). Edit-ON and edit-OFF transients were added to yield the SUM spectra. Average GABA-DIFF and SUM metabolite spectra were modelled using a TE-specific simulated basis set and a flexible spline baseline available on Osprey and based on MRS vendor, pulse duration, and scan sequence parameters (Oeltzschner et al., 2020; Simpson et al., 2017). Basis sets for macromolecule and lipid contributions were integrated as Gaussian basis functions (Oeltzschner et al., 2020). Difference and SUM spectra were modelled between 0.5 ppm and 4 ppm with linear baseline correction and a knot spacing of 0.55 ppm according to the Osprey model algorithm (Oeltzschner et al., 2020).

**Fig. 2.**
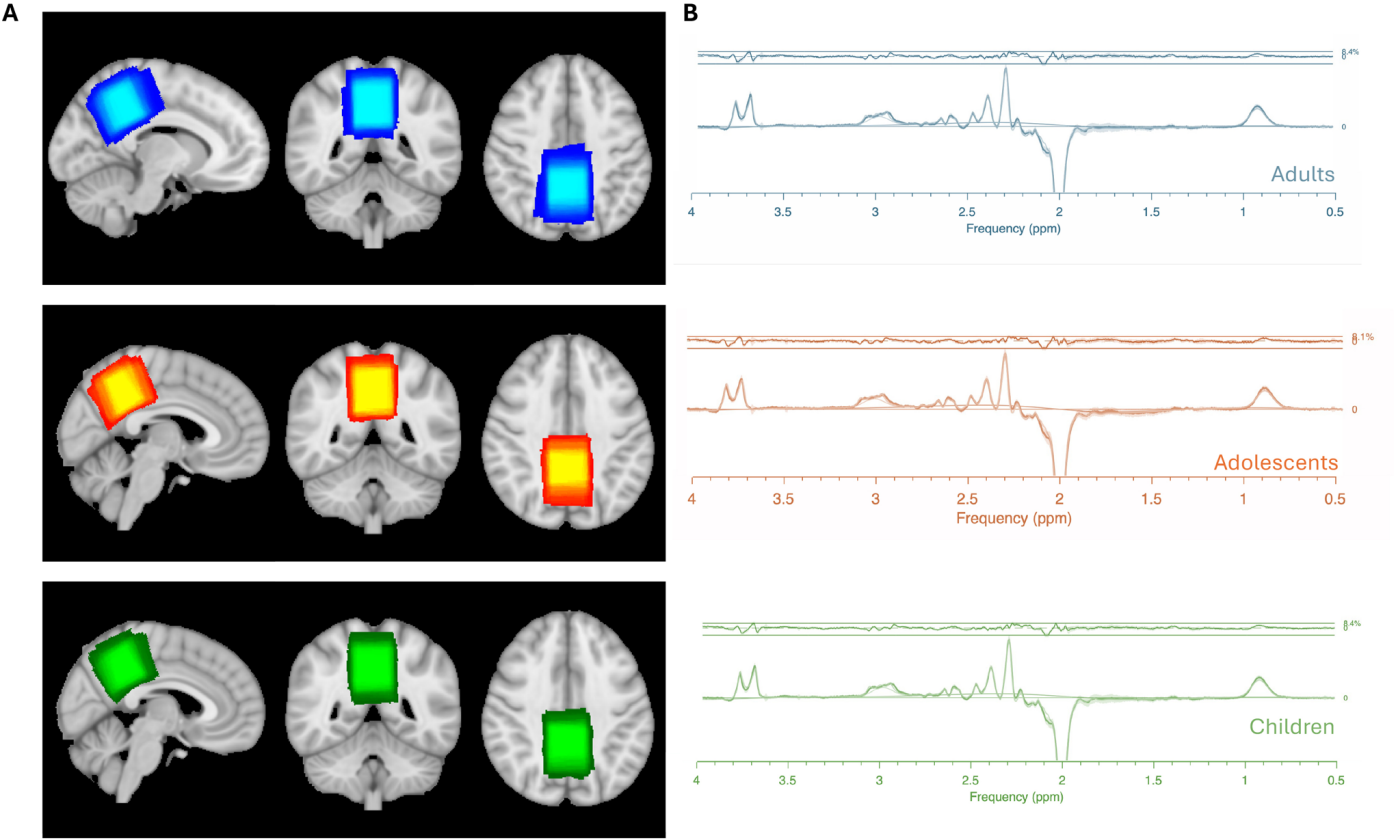
(A) Standard T_1_-weighted image displaying overlap heat maps of MRS voxels in the posterior parietal cortex across age groups: adults (blue), adolescents (orange), and children (green). Overlap plots were generated by registering individual T_1_-weighted images and voxel masks to a standard space (MNI152 T_1_-weighted 1 mm brain) and computing the voxel overlap. The resulting heat maps are overlaid on the standard anatomical template. (B) Mean MEGA-PRESS GABA-DIFF spectra per age group, showing model fit, baseline, and median residuals (error bars). Ribbon plots indicate the standard deviation of raw spectra fit to the basis sets. Mean SUM spectra are shown in Supplementary Figure 1.

GABA+ was quantified in the difference spectra, while total NAA (tNAA), total Cho (tCho), total Cr (tCr), mI, and Glx were quantified in the SUM spectra. Note GABA signals are quantified as GABA+ due to contamination from overlapping macromolecular signals, while glutamate is commonly reported as Glx (a combined measure of glutamate and its precursor glutamine) due to significant spectral overlap (Harris et al., 2017; Mullins et al., 2014; Puts & Edden, 2012). Average GABA-difference spectra are shown in Figure 2 and average SUM spectra are shown in Supplementary Figure 1. The Osprey co-registration module (via SPM version 12; (Friston et al., 2007)) was used to register MRS to the T_1_-weighted images acquired at the scan, and segment the voxel volume into grey matter fraction (fGM), white matter fraction (fWM), and cerebrospinal fluid fraction (fCSF). Outputs were visually inspected to ensure accurate localisation and segmentation of the MRS voxel. Segmented T_1_-weighted images were used to obtain tissue-composition-corrected water-scaled estimates of neuro-metabolite concentrations in institutional units (IU), using three approaches: first, water-scaled concentrations were scaled according to the assumption that neuro-metabolite concentrations in CSF are negligible (Gasparovic et al., 2006; *CSF-corrected metabolite levels*). Second, water-scaled metabolite concentrations were corrected for differences in metabolite and water T_1_ and T_2_ relaxation times, as well as for differences in water content across GM, WM, and CSF, following established methods (Gasparovic et al., 2006; Oeltzschner et al., 2020; *Tissue-corrected metabolite levels*). Relaxation values used for tissue correction are reported in Supplementary Table 2. Third, “alpha correction” of *tissue-corrected* GABA+ concentrations was performed, with the assumption that GABA+ concentration is two times more concentrated in GM than in WM (Harris et al., 2015; Jensen et al., 2005; *alpha-corrected GABA+ levels*). Neuro-metabolite concentrations are not reported relative to total creatine (e.g. tCho/tCr; creatine-scaled), as in our previous work we show that creatine levels are unstable across the developmental period studied (Thomson, Hwa, et al., 2024). Instead, we focus interpretations on estimated *tissue-corrected* metabolite concentrations (and *alpha corrected* for GABA+), which we report in IU. *CSF-corrected* metabolite analyses are also reported in the Supplementary Materials to observe potential tissue-correction biases. T_1_-weighted images and voxel masks were registered to a standard MN1152 T_1_-weighted 1 mm brain anatomical image for the creation of voxel heat plot shown in Figure 2.

### 2.5 Statistical analysis

Analysis was performed in R (Version 4.2.2) using the *lm* function. To observe the effect of neuro-metabolite concentrations on PPC voxel structural metrics, linear regression was performed with neuro-metabolite concentration, sex, age, IQ, frequency shift (Hz), and estimated total intracranial volume (eTIV) as predictors and the PPC voxel structural metric as the dependent variable (structural metric ~ neuro-metabolite + age + IQ + sex + frequency shift + eTIV). In doing so, we aimed to observe associations between structure and neuro-metabolites and potential effects of IQ, sex, and age, while controlling for data quality (frequency shift) and whole-brain volume. Controlling for eTIV is common practice for regional brain volume and cortical thickness association studies (see section 4; Backhausen et al., 2022; Jiang et al., 2016; Voevodskaya et al., 2014). In our data, eTIV showed no significant collinearity with age (beta = 1594.00, p = 0.49), supporting its inclusion in the model. Note that, for surface area metrics, mean cortical surface area was used in place of eTIV in regression models. Sex–age interactions were included in each model also. IQ was included as this has previously been associated with cortical thickness variation (Menary et al., 2013). Frequency shift was added as a proxy for movement effects in MRS data (Saleh et al., 2020). Partial residual plots to reflect the effect of neuro-metabolite concentration on voxel structural metrics (with age, sex, IQ eTIV, and frequency shift held constant) were created using the *effects* package function (Fox & Weisberg, 2011, 2018).

As some neuro-metabolite levels have previously been shown to change non-linearly with age (Thomson, Hwa, et al., 2024), the associations between voxel structural metrics and neuro-metabolite concentrations were also modelled using generalised additive models (GAM’s). In these models, age was included as a non-linear smooth Gaussian process term, while neuro-metabolite concentrations, eTIV, frequency shift, IQ, and sex were included as parametric predictors (structural metric ~ neuro-metabolite + s(age) + IQ + sex + eTIV + frequency shift). The Restricted Maximum Likelihood (REML) method was used to select smoothing parameters and model adequacy was evaluated using the “gam.check” function (Augustin et al., 2012), ensuring that residuals were randomly distributed. For GAM, the effective degrees of freedom (edf) of each smooth function represents the complexity of the smoothing. Edf’s of 1 indicate a linear relationship between the dependent variable and predictor, while edfs of 2 indicate a quadratic relationship; greater edf values as such indicate a greater degree of non-linearity. Reference degrees of freedom and the F statistic were used to assess the significance of predictor smooth functions (significance meaning greater certainty of the degree of smoothness; see Thomson, Hwa, et al., 2024).

To correct for the multiple comparisons during regression and GAM analysis of metabolite–structural associations (15 neuro-metabolites per dependent variable), False Discovery Rate (FDR) correction was applied. FDR controls for the expected proportion of false positives (5% of significant findings). FDR correction also accounts for potential dependencies among statistical tests, a condition that is likely to hold in this case, as PPC neuro-metabolite concentrations are metabolically associated (Rae, 2014). Statistical significance was assessed at an adjusted p-value threshold of p_adjusted_ < 0.05, with exact FDR-adjusted p-values reported.

## 3 Results

### 3.1 Associations between PPC voxel structural metrics and neuro-metabolite levels

Age-related changes in neuro-metabolite concentrations, previously reported in Thomson, Hwa, et al. (2024), are shown in Supplementary Figure 2. To assess the consistency with previously characterised age-related changes in cortical macrostructure (Baik et al., 2023; Ducharme et al., 2016; Frangou et al., 2022; Koolschijn & Crone, 2013; Muftuler et al., 2011), we evaluated whether our PPC voxel macrostructural metrics associated with age. In linear regressions, age significantly and negatively associated with PPC voxel mean cortical thickness, cortical volume, cortical area, and LGI when controlling for eTIV (Supplementary Fig. 3 & Supplementary Table 3). Age significantly interacted with sex to predict mean voxel cortical thickness and mean voxel volume; however age-related declines were similar between sexes (Supplementary Fig. 4). In a separate GAM analysis, we found that age-related declines were also significantly non-linear for all voxel metrics (Supplementary Table 4), this was true for both sexes.

Next, we evaluated associations between PPC voxel structural metrics and neuro-metabolite levels using linear regressions and GAMs, which controlled for eTIV, sex, IQ, age (as a non-linear term in GAMs), and frequency shift. Note all extracted model statistics (beta coefficients, p values, edf values, and R squared (R²)) are reported in Supplementary Tables 5–12.

**Fig. 3.**
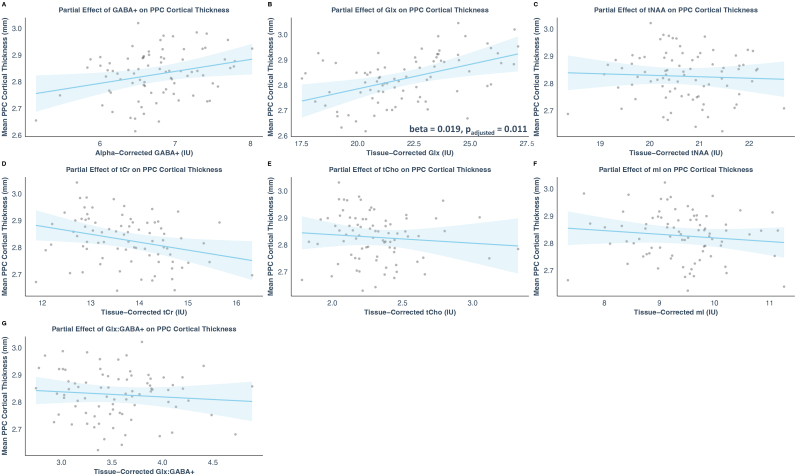
(A-G) PPC cortical thickness. Partial linear regression plot shows cortical thickness (mm) predicted by tissue-corrected neuro-metabolite concentration (IU) holding participant age, sex, IQ, frequency shift, and eTIV constant. Blue shading represents the 95% confidence interval for the partial regression prediction. Points represent individual partial residuals (effect of age, sex, IQ, frequency shift, and eTIV regressed out). Significant beta coefficient estimates are shown.

**Fig. 4.**
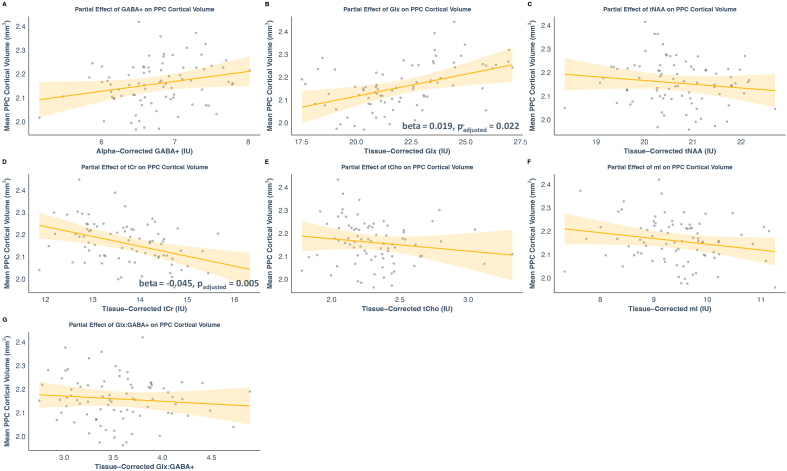
(A-G) PPC cortical volume. Partial linear regression plot of PPC voxel mean cortical volume (mm^3^) predicted by tissue-corrected neuro-metabolite concentration (IU) holding participant age, sex, IQ, frequency shift, and eTIV constant. Yellow shading represents the 95% confidence interval for the partial regression prediction. Points represent individual partial residuals. Significant beta coefficient estimates are shown.

**Fig. 5.**
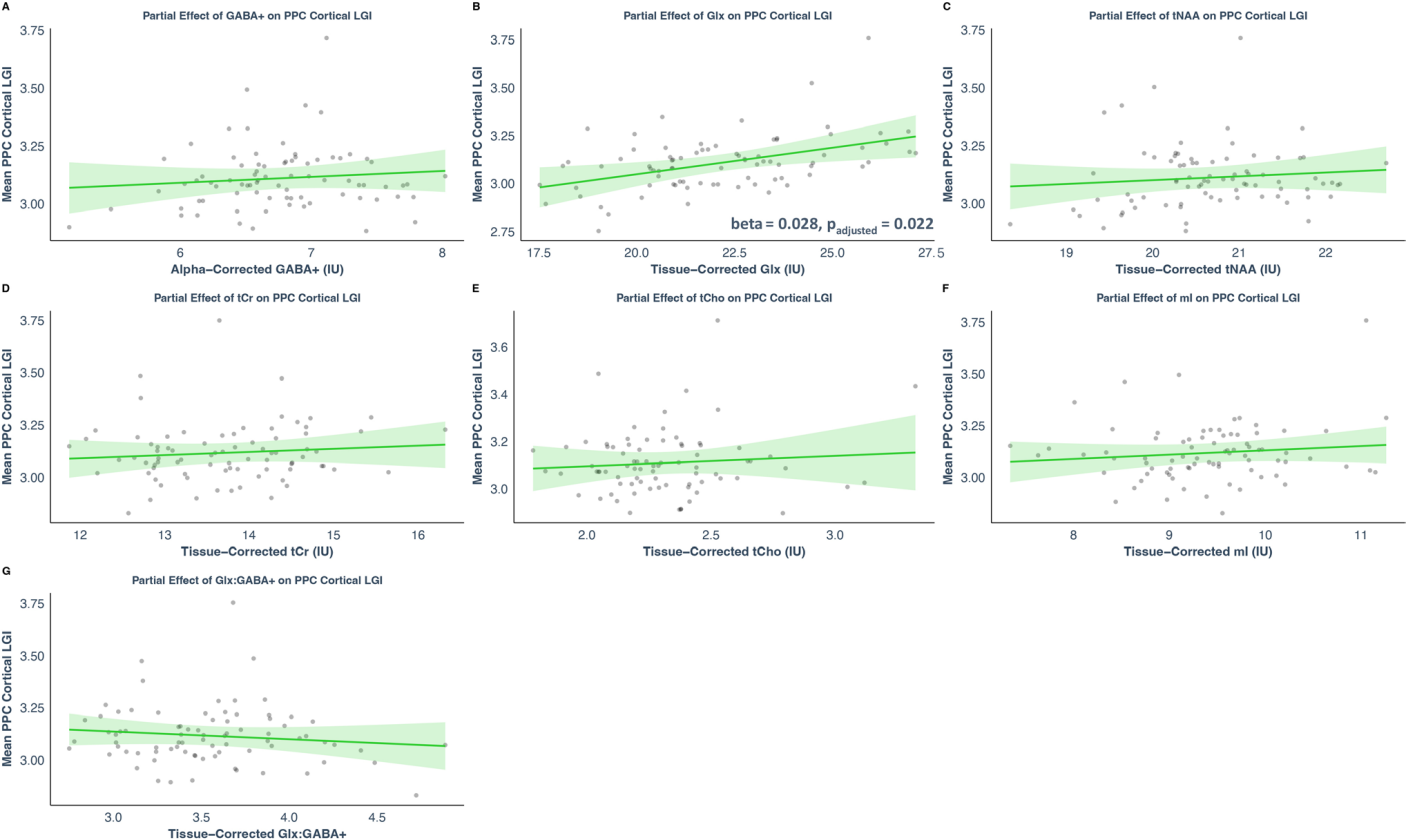
(A-G) PPC LGI. Partial linear regression plot of PPC voxel cortical LGI ratio predicted by tissue-corrected neuro-metabolite concentration (IU) holding participant age, sex, IQ, frequency shift, and eTIV constant. Green shading represents the 95% confidence interval for the partial regression prediction. Points represent individual partial residuals. Significant beta coefficient estimates are shown.

In linear regressions, a significant and positive linear association was observed between tissue-corrected Glx and PPC voxel mean cortical thickness (beta = 0.019, p_adjusted_ = 0.011; Fig. 3; Supplementary Table 5), as well as PPC voxel mean cortical volume (beta = 0.019, p_adjusted_ = 0.022; Fig. 4; Supplementary Table 9). However, these associations were not significant when age was included as a non-linear predictor in GAMs (Supplementary Table 6 and Supplementary Table 10). Tissue-corrected Glx also significantly and positively associated with mean PPC voxel LGI, both in the linear regression where age was added as a linear term (beta = 0.028, p_adjusted_ = 0.022; Fig. 5; Supplementary Table 11) and in the GAM where age was included as a non-linear term (beta = 0.028, p_adjusted_ = 0.019; Supplementary Table 12).

**Fig. 6.**
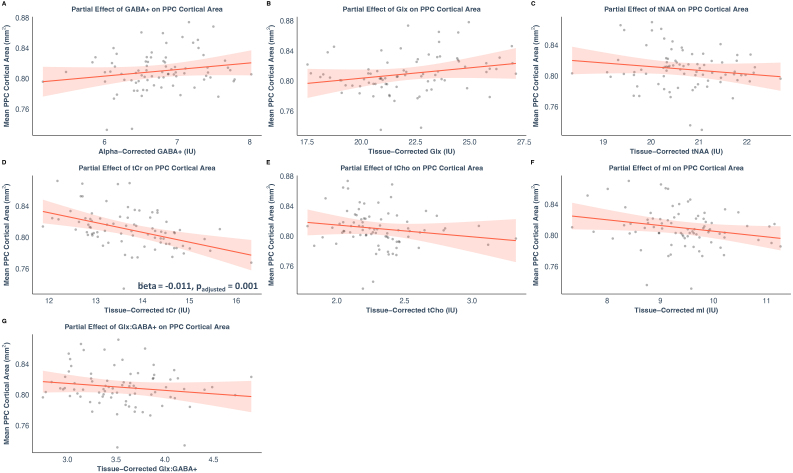
(A-G) PPC cortical area. Partial linear regression plot of PPC voxel mean cortical area (mm^2^) predicted by tissue-corrected neuro-metabolite concentration (IU) holding participant age, sex, IQ, frequency shift, and eTIV constant. Red shading represents the 95% confidence interval for the partial regression prediction. Points represent individual partial residuals. Significant beta coefficient estimates are shown.

Using linear regression, tissue-corrected tCr significantly and negatively associated with PPC voxel mean cortical area (beta = -0.011, p_adjusted_ = 0.001; Fig. 6; Supplementary Table 7) and PPC voxel mean cortical volume (beta = -0.045, p_adjusted_ = 0.005; Fig. 4; Supplementary Table 9). These associations were also observed when age was included as a non-linear term in GAMs for voxel mean cortical area (beta = -0.009, p_adjusted_ = 0.019; Supplementary Table 8) and volume (beta = -0.028, p_adjusted_ = 0.042; Supplementary Table 10). Note associations between PPC voxel bulk tissue content (GM, WM, and CSF) and neuro-metabolite levels are reported in Supplementary Figure 5. In all metabolite–structural metric models (linear regression and GAM), no significant interactions between sex and age or significant independent effect of IQ or sex was found (Supplementary Tables 5–12).

## 4 Discussion

In this study, we present the first investigation of the associations between MRS-measured neuro-metabolite concentrations and cortical macrostructural metrics in a PPC voxel obtained from a developmental cohort spanning 5–35 years of age. Our findings provide novel insights into the neuroanatomical correlates of changes in brain chemistry across childhood to early adulthood, with implications for the interpretation of MRS-measured neuro-metabolite levels, and their macrostructural links, in both typical and atypical brain development.

### 4.1 PPC voxel cortical thickness, area, and volume

First we confirmed that cortical thickness, area, and volume in the PPC decrease across childhood to early adulthood, consistent with cortical thinning observed in the parietal cortex and multiple other cortical regions across the same period (Baik et al., 2023; Ducharme et al., 2016; Frangou et al., 2022; Koolschijn & Crone, 2013; Muftuler et al., 2011). We show that PPC cortical thickness and volume positively associated with Glx concentrations across development. Previous studies in adolescence and adulthood report no significant reductions in cortical neuron density (Freeman et al., 2008; la Fougère et al., 2011), despite this, both cortical thickness and Glx decrease across the period studied (Thomson, Pasanta, et al., 2024). Thus, individual differences in neuron density are likely not the sole driver of this association (Freeman et al., 2008; la Fougère et al., 2011). Instead, these findings may reflect neuronal and glial cell microarchitectural differences that contribute to individual variability in both cortical macrostructure and Glx across development (Sailasuta et al., 2008; Thomson, Hwa, et al., 2024).

More specifically, histological studies link cortical thinning to changes in neuronal dendritic arbour complexity, which is remodelled by synaptic pruning across the period studied as the cortex undergoes circuit refinement (Frangou et al., 2022; Huttenlocher, 1979; Huttenlocher & Dabholkar, 1997; Madre et al., 2020; Petanjek et al., 2011). Gene association studies support this, linking genes involved in dendritic spine formation and stability in cortical pyramidal neurons to regional variations in cortical thickness (Parker et al., 2020; Shin et al., 2018; Z. Zhou et al., 2023). Dendritic spines, small protrusions on the dendritic shaft where the majority of excitatory synapses in the cortex are located (Amaral & Pozzo-Miller, 2009), are primary sites of developmental synaptic pruning (Petanjek et al., 2011). Given that glutamate, the brain’s primary excitatory neurotransmitter, plays central roles in synaptic activity at dendritic spines, Glx concentrations are likely also related to variations in dendritic arborisation and spine stability. This is supported by findings that Glx concentrations positively correlate with local synaptic density, estimated using positron emission tomography (PET) to detect radiolabelled synaptic vesicle glycoproteins (SV2A; Onwordi et al., 2021). As such, individual differences in dendritic arbour complexity, mediated by synaptic pruning across the developmental period studied, likely contribute to differences in both Glx concentrations and cortical thickness and so the observed association between these traits.

Furthermore, synaptic pruning mechanisms have been linked to glutamatergic signalling (Cachope & Pereda, 2021; Lohmann & Kessels, 2013; Narushima et al., 2016; Surdin et al., 2023). For example, microglia cells engulf synaptic components for pruning, their activity regulated by metabotropic glutamate receptors (Bie et al., 2019; Kim et al., 2017). Given that genes expressed in microglia have also been implicated in cortical thickness variation (Parker et al., 2020; Shin et al., 2018; Zhou et al., 2023), it is plausible that Glx concentrations regulate microglial synapse elimination processes on dendritic arbours, thereby driving cortical thickness variation. Further research is required to evidence this and to understand how MRS neurochemical dynamics influence or are influenced by micro- and macrostructural brain differences.

Note that cortical thickness differences have also been linked to the myelination of deep intracortical fibres. Cortical thinning during childhood and adolescence is parallel with widespread increases in the fractional anisotropy of major white matter tracts, indicative of protracted nerve fibre myelination and alignment (Coll et al., 2020; Grydeland et al., 2013; Kinney et al., 1988; Lebel et al., 2008). Myelination may alter the MR signal intensity of deep cortical layers, contributing to age-related reductions in cortical thickness estimations (Miller et al., 1996, 2012; Natu et al., 2019; Paus et al., 2008; Veenstra-VanderWeele et al., 2017; Vidal-Pineiro et al., 2020). Given the positive relationship between Glx concentrations and voxel GM volume, individual differences in deep layer myelination may also contribute to the association between cortical thickness and Glx across the period studied (Zhang & Shen, 2015).

While the mechanisms underlying cortical area differences are less understood (Norbom et al., 2021), we find a negative association between PPC cortical area and creatine levels. The lack of a Glx–cortical area association, combined with the specific link between creatine and cortical area, suggests that distinct mechanisms govern cortical area and thickness in childhood and adolescence, as is supported by previous neuroimaging and genetic studies (Grasby et al., 2020; Norbom et al., 2021; van der Meer et al., 2020). Furthermore, given creatines’ role in cellular energy buffering (creatine–phosphocreatine flux; Grasby et al., 2020; Rae, 2014; Wallimann et al., 1992), mechanisms that drive cortical area variation are suggestively more energetic. Since cortical volume reflects both thickness and area, it is likely shaped by a combination of genetic and environmental factors affecting these traits (Norbom et al., 2021). Accordingly, cortical volume shows significant associations with both tCr (through area-related effects) and Glx (through thickness-related effects).

### 4.2 PPC voxel LGI

We find that PPC cortical LGI significantly decreased across the developmental period studied. Changes in LGI were notably more gradual than changes in cortical thickness, consistent with findings in similar developmental cohorts in frontal and temporal regions (Klein et al., 2014; White et al., 2010). The folding or gyrification of the brain predominantly occurs prenatally but continues to adulthood, while the LGI ratio itself becomes relatively stable from the first year of life after birth (White et al., 2010). Gyrification is thought to be essential for the creation of efficient neural networks by reducing the distance between neighbouring brain regions to increase communication speeds (White et al., 2010). We find that PPC cortical LGI, similar to cortical thickness and volume, is positively associated with tissue-corrected Glx. Glutamatergic signalling has roles in neuronal migration and axon pathfinding, specifically tuning neuronal responses to axon guidance cues (Kaindl et al., 2008; Luhmann et al., 2015; Tran et al., 2024). Such processes are fundamental to developing early structural connectivity which, in turn, influences cortical folding forces and patterns (Ecker et al., 2016; Nie et al., 2012; Ouyang et al., 2023; Ronan et al., 2014). While mechanisms underlying the subtle changes in LGI observed in our study likely differ from those related to the establishment of gyrification during the prenatal and early post-natal period (Hasan et al., 2011), this association between LGI and Glx might reflect differences in gyrification which were established and associated with glutamatergic mechanisms early in development and remain detectable in the study period.

Alternatively, changes in LGI during childhood and adolescence have previously been associated with the same mechanisms underpinning changes in cortical thickness. This includes dendritic remodelling (White et al., 2010) and intra-cortical fibre myelination, with the diffusivity of white matter fibre tracts specifically associated with individual differences in regional LGI (Ecker et al., 2016). Individual microstructural differences across development may thus alter folding forces, leading to cortical “flattening” observed across the period studied, as well the association between LGI and Glx (Ecker et al., 2016; White et al., 2010).

### 4.3 Age-dependent effects

In our previous work (Thomson, Hwa, et al., 2024), we demonstrated that some neuro-metabolite trajectories across the lifespan are non-linear. To account for these non-linear developmental patterns, we additionally incorporated age as a non-linear predictor in Generalised Additive Models (GAMs) to further examine associations between neuro-metabolite levels and PPC voxel structural metrics. The majority of significant associations observed in linear regression models (with age as a linear term) remained significant in the GAMs, with two exceptions: associations between tissue-corrected Glx and mean PPC voxel cortical volume and mean PPC voxel cortical thickness were no longer statistically significant. This indicates that these Glx–macrostructure associations are largely explained by age-related developmental processes (described above), with brain maturation driving parallel changes in local Glx, cortical volume, and thickness. In contrast, the persistence of the negative association between creatine and cortical area, and the positive association between Glx and cortical LGI in the non-linear models suggest that these relationships are potentially independent of age and instead reflect inter-individual variability.

## 5 Limitations

Tissue-corrected neuro-metabolite concentrations, which account for individual differences in voxel tissue composition (GM, WM, and CSF), were used in this study as recommended (Gasparovic et al., 2006; Lin et al., 2021; Peek et al., 2023). This involves applying corrections for tissue-specific water and neuro-metabolite relaxation differences (Gasparovic et al., 2006; Lin et al., 2021; Near et al., 2011); however, we acknowledge that these values are derived from adult populations (Oeltzschner et al., 2020). In younger populations, particularly children, neuro-metabolite-specific relaxation times remain largely undefined. Given that both water and neuro-metabolite (longitudinal and transverse) relaxation times are thought to vary with age, this introduces uncertainty in neuro-metabolite quantification across the lifespan. Ideally, relaxation times would be empirically measured for each neuro-metabolite, per participant or age group, to ensure more accurate tissue correction and quantification. However, this would require significantly extended scan times or additional scanning sessions, as relaxation parameters must be acquired separately for each metabolite and sequence. As a result, this approach was not logistically feasible within the study design. While the magnitude of age-related changes in relaxation times may be relatively modest (Kirov et al., 2008; Mlynárik et al., 2001, 2006; Murali-Manohar et al., 2025; Puts, Barker, et al., 2013), their potential to introduce systematic bias, particularly in developmental studies, should not be overlooked. Future work should aim to define metabolite-specific relaxation parameters in paediatric and adolescent populations to improve the accuracy and interpretability of lifespan MRS research.

For this reason, we refrain from reporting tissue-corrected neuro-metabolite concentrations as “absolute concentrations” in millimoles (mM) or micromoles per gram (µmol/g), to avoid suggesting a level of quantification precision that may not be supported. Several additional factors, such as inter-scanner variability, B_0_ inhomogeneity, and macromolecular contamination can also influence neuro-metabolite signal amplitudes in ways that are not fully accounted for. For this reason, we chose to report values in institutional units (IU). Nonetheless, our quantification pipeline followed best-practice recommendations (Gasparovic et al., 2006; Lin et al., 2021; Near et al., 2011), and we have provided clear detail on the correction methods used to facilitate future comparisons.

The accuracy of voxel tissue segmentation and FreeSurfer-derived cortical surface metrics may also be influenced by age, as segmentation algorithms were originally trained on adult datasets (Cabezas et al., 2011; Harris et al., 2015). However, FreeSurfer has demonstrated reliable performance in tissue segmentation even in young children, including MRI data from 5-year-old children (Pulli et al., 2022). Moreover, our key findings were replicated in analyses using neuro-metabolite data that were uncorrected for voxel tissue composition (i.e., corrected only for CSF; see Supplementary Materials), which are less reliant on accurate segmentation of grey and white matter. This partial replication supports the robustness of the identified associations despite potential age-related segmentation biases.

For all analyses, estimated total intracranial volume (eTIV) was included as a covariate to account for age-related changes in brain volume. Significant associations identified between neuro-metabolite concentrations and structural metrics were, therefore, independent of overall brain volume differences (Backhausen et al., 2022; Jiang et al., 2016; Voevodskaya et al., 2014). Alternative approaches include the use of normalised structural measures (e.g., dividing structural metrics by eTIV), which we chose not to use for several reasons. First, normalisation approaches vary significantly across studies, complicating the interpretation of findings. Second, our aim was to investigate the associations between PPC neuro-metabolite concentrations and structural metrics. Normalisation might have shifted the focus towards deviations in PPC structure relative to whole-brain averages, obscuring the relationships of interest. Finally, all age-related trends observed in our raw structural metrics (when eTIV was controlled for statistically) are consistent with previous work (Baik et al., 2023; Ducharme et al., 2016; Frangou et al., 2022; Koolschijn & Crone, 2013; Muftuler et al., 2011). However, we acknowledge that alternative approaches may provide complementary perspectives depending on the research focus.

To account for potential motion-related artefacts in edited-MRS data, frequency shift was also included as a covariate in all analyses (Saleh et al., 2020). Although motion is presumed to be more prevalent in younger participants, this was not supported by findings from Puts, Mikkelsen, et al., 2017, and consistent with this, we observed comparable MRS data exclusion rates between children and adults. All included MRS datasets passed rigorous quality control procedures to ensure good data quality. Nevertheless, it is important to acknowledge that MRS inherently exhibits greater signal variance compared with structural MRI, largely due to the relatively low brain concentration of metabolites. Despite this, all significant associations reported in our analyses survived correction for multiple comparisons, indicating robust and reliable effects. Moreover, the linear regression models consistently demonstrated high explanatory power (R² ~ 0.5–0.8), with no significant effects observed for the covariates sex, IQ, or frequency shift. Together, these findings highlight the significant contribution of structural brain measures to variability in neuro-metabolite concentrations and support the robustness of our results.

## 6 Conclusion

This study provides novel insights into the relationship between MRS-measured neuro-metabolite concentrations and cortical macrostructure across development. We show age-dependent associations between PPC Glx and local cortical thickness, volume, and LGI, likely because differences in cortical microstructure, including dendritic arbour complexity, contribute to differences in cortical macrostructural traits and Glx activity across development. We further show age-independent associations between PPC creatine and local cortical surface area and volume, suggesting that inter-individual variability in these cortical macrostructures is linked to underlying tissue energetic properties. These findings underscore the importance of accounting for macrostructural variation when interpreting MRS data, especially when comparing neuro-metabolite concentrations across groups with brain structural differences, such as in neurodevelopmental disorders (Ecker et al., 2022; Hardan et al., 2006; Hyde et al., 2010; You et al., 2024) or across age. Future research should further explore the interdependencies between neurochemical and macrostructural changes, to determine whether neuro-metabolite alterations are driven by structural changes or whether the chemical alterations themselves drive structural changes.

## Supplementary Material

Supplementary Material

## Data Availability

Data are available through: https://osf.io/uv67s/?view_only=2896964f249f4249bbfdeccb0b96bb73. Osprey 2.4.0 is available through: https://github.com/schorschinho/osprey

## References

[IMAG.a.1041-b1] Alemán-Gómez, Y., Janssen, J., Schnack, H., Balaban, E., Pina-Camacho, L., Alfaro-Almagro, F., Castro-Fornieles, J., Otero, S., Baeza, I., Moreno, D., Bargalló, N., Parellada, M., Arango, C., & Desco, M. (2013). The human cerebral cortex flattens during adolescence. The Journal of Neuroscience, 33(38), 15004–15010. 10.1523/JNEUROSCI.1459-13.2013 24048830 PMC6618418

[IMAG.a.1041-b2] Amaral, M. D., & Pozzo-Miller, L. (2009). The dynamics of excitatory synapse formation on dendritic spines. Cellscience, 5(4), 19–25. 10.1016/b978-012370509-9.00039-5 20072712 PMC2805008

[IMAG.a.1041-b3] Augustin, N., Sauleau, E.-A., & Wood, S. (2012). On quantile quantile plots for generalized linear models. Computational Statistics & Data Analysis, 56(8), 2404–2409. 10.1016/j.csda.2012.01.026

[IMAG.a.1041-b4] Backhausen, L. L., Herting, M. M., Tamnes, C. K., & Vetter, N. C. (2022). Best practices in structural neuroimaging of neurodevelopmental disorders. Neuropsychology Review, 32(2), 400–418. 10.1007/s11065-021-09496-2 33893904 PMC9090677

[IMAG.a.1041-b5] Baik, K., Jeon, S., Yang, S.-J., Na, Y., Chung, S. J., Yoo, H. S., Yun, M., Lee, P. H., Sohn, Y. H., & Ye, B. S. (2023). Cortical thickness and brain glucose metabolism in healthy aging. Journal of Clinical Neurology (Seoul, Korea), 19(2), 138–146. 10.3988/jcn.2022.0021 36647225 PMC9982173

[IMAG.a.1041-b6] Bie, B., Wu, J., Foss, J. F., & Naguib, M. (2019). Activation of mGluR1 mediates C1q-dependent microglial phagocytosis of glutamatergic synapses in Alzheimer’s rodent models. Molecular Neurobiology, 56(8), 5568–5585. 10.1007/s12035-019-1467-8 30652266 PMC6615956

[IMAG.a.1041-b7] Bourgeois, J. P., Goldman-Rakic, P. S., & Rakic, P. (1994). Synaptogenesis in the prefrontal cortex of rhesus monkeys. Cerebral Cortex (New York, N.Y.: 1991), 4(1), 78–96. 10.1093/cercor/4.1.78 8180493

[IMAG.a.1041-b8] Bourgeois, J. P., Jastreboff, P. J., & Rakic, P. (1989). Synaptogenesis in visual cortex of normal and preterm monkeys: Evidence for intrinsic regulation of synaptic overproduction. Proceedings of the National Academy of Sciences of the United States of America, 86(11), 4297–4301. 10.1073/pnas.86.11.4297 2726773 PMC287439

[IMAG.a.1041-b9] Cabezas, M., Oliver, A., Lladó, X., Freixenet, J., & Cuadra, M. B. (2011). A review of atlas-based segmentation for magnetic resonance brain images. Computer Methods and Programs in Biomedicine, 104(3), e158–177. 10.1016/j.cmpb.2011.07.015 21871688

[IMAG.a.1041-b10] Cachope, R., & Pereda, A. E. (2021). Regulatory roles of metabotropic glutamate receptors on synaptic communication mediated by gap junctions. Neuroscience, 456, 85–94. 10.1016/j.neuroscience.2020.06.034 32619474 PMC7805574

[IMAG.a.1041-b11] Coll, G., de Schlichting, E., Sakka, L., Garcier, J.-M., Peyre, H., & Lemaire, J.-J. (2020). Assessment of maturational changes in white matter anisotropy and volume in children: A DTI study. AJNR. American Journal of Neuroradiology, 41(9), 1726–1732. 10.3174/ajnr.A6709 32816761 PMC7583127

[IMAG.a.1041-b12] Dale, A. M., Fischl, B., & Sereno, M. I. (1999). Cortical surface-based analysis. I. Segmentation and surface reconstruction. NeuroImage, 9(2), 179–194. 10.1006/nimg.1998.0395 9931268

[IMAG.a.1041-b13] Ducharme, S., Albaugh, M. D., Nguyen, T.-V., Hudziak, J. J., Mateos-Pérez, J. M., Labbe, A., Evans, A. C., Karama, S., & Brain Development Cooperative Group. (2016). Trajectories of cortical thickness maturation in normal brain development—The importance of quality control procedures. NeuroImage, 125, 267–279. 10.1016/j.neuroimage.2015.10.010 26463175 PMC4691414

[IMAG.a.1041-b14] Ecker, C., Andrews, D., Dell’Acqua, F., Daly, E., Murphy, C., Catani, M., Thiebaut de Schotten, M., Baron-Cohen, S., Lai, M. C., Lombardo, M. V., Bullmore, E. T., Suckling, J., Williams, S., Jones, D. K., Chiocchetti, A., & Murphy, D. G. M. (2016). Relationship between cortical gyrification, white matter connectivity, and autism spectrum disorder. Cerebral Cortex (New York, NY), 26(7), 3297–3309. 10.1093/cercor/bhw098 PMC4898679 27130663

[IMAG.a.1041-b15] Ecker, C., Pretzsch, C. M., Bletsch, A., Mann, C., Schaefer, T., Ambrosino, S., Tillmann, J., Yousaf, A., Chiocchetti, A., Lombardo, M. V., Warrier, V., Bast, N., Moessnang, C., Baumeister, S., Dell’Acqua, F., Floris, D. L., Zabihi, M., Marquand, A., Cliquet, F., … Murphy, D. G. M. (2022). Interindividual differences in cortical thickness and their genomic underpinnings in autism spectrum disorder. American Journal of Psychiatry, 179(3), 242–254. 10.1176/appi.ajp.2021.20050630 34503340

[IMAG.a.1041-b16] Edden, R. A. E., Oeltzschner, G., Harris, A. D., Puts, N. A. J., Chan, K., Boer, V. O., Schär, M., & Barker, P. B. (2016). Prospective frequency correction for macromolecule suppressed GABA editing experiments at 3T. Journal of Magnetic Resonance Imaging: JMRI, 44(6), 1474–1482. 10.1002/jmri.25304 27239903 PMC5118053

[IMAG.a.1041-b17] Faust, T. E., Gunner, G., & Schafer, D. P. (2021). Mechanisms governing activity-dependent synaptic pruning in the developing mammalian CNS. Nature Reviews Neuroscience, 22(11), 657–673. 10.1038/s41583-021-00507-y 34545240 PMC8541743

[IMAG.a.1041-b18] Fischl, B., & Dale, A. M. (2000). Measuring the thickness of the human cerebral cortex from magnetic resonance images. Proceedings of the National Academy of Sciences of the United States of America, 97(20), 11050–11055. 10.1073/pnas.200033797 10984517 PMC27146

[IMAG.a.1041-b19] Fischl, B., Sereno, M. I., & Dale, A. M. (1999). Cortical surface-based analysis. II: Inflation, flattening, and a surface-based coordinate system. NeuroImage, 9(2), 195–207. 10.1006/nimg.1998.0396 9931269

[IMAG.a.1041-b20] Fleming, L. L., & McDermott, T. J. (2024). Cognitive control and neural activity during human development: Evidence for synaptic pruning. Journal of Neuroscience, 44(26), e0373242024. 10.1523/JNEUROSCI.0373-24.2024 38926080 PMC11211714

[IMAG.a.1041-b21] Fox, J., & Weisberg, S. (2011). An R companion to applied regression. SAGE. 10.32614/cran.package.car

[IMAG.a.1041-b22] Fox, J., & Weisberg, S. (2018). Visualizing fit and lack of fit in complex regression models with predictor effect plots and partial residuals. Journal of Statistical Software, 87, 1–27. 10.18637/jss.v087.i09

[IMAG.a.1041-b23] Frangou, S., Modabbernia, A., Williams, S. C. R., Papachristou, E., Doucet, G. E., Agartz, I., Aghajani, M., Akudjedu, T. N., Albajes-Eizagirre, A., Alnaes, D., Alpert, K. I., Andersson, M., Andreasen, N. C., Andreassen, O. A., Asherson, P., Banaschewski, T., Bargallo, N., Baumeister, S., Baur-Streubel, R., … Dima, D. (2022). Cortical thickness across the lifespan: Data from 17,075 healthy individuals aged 3–90 years. Human Brain Mapping, 43(1), 431–451. 10.1002/hbm.25364 33595143 PMC8675431

[IMAG.a.1041-b24] Freeman, S. H., Kandel, R., Cruz, L., Rozkalne, A., Newell, K., Frosch, M. P., Hedley-Whyte, E. T., Locascio, J. J., Lipsitz, L., & Hyman, B. T. (2008). Preservation of neuronal number despite age-related cortical brain atrophy in elderly subjects without Alzheimer disease. Journal of Neuropathology and Experimental Neurology, 67(12), 1205–1212. 10.1097/NEN.0b013e31818fc72f 19018241 PMC2734185

[IMAG.a.1041-b25] Friston, K., Ashburner, J., Kiebel, S., & Nichols, T. (2007). Statistical parametric mapping: The analysis of functional brain images. https://www.bdi.ox.ac.uk/publications/908842

[IMAG.a.1041-b26] Gasparovic, C., Song, T., Devier, D., Bockholt, H. J., Caprihan, A., Mullins, P. G., Posse, S., Jung, R. E., & Morrison, L. A. (2006). Use of tissue water as a concentration reference for proton spectroscopic imaging. Magnetic Resonance in Medicine, 55(6), 1219–1226. 10.1002/mrm.20901 16688703

[IMAG.a.1041-b27] Geschwind, D. H. (2011). Genetics of autism spectrum disorders. Trends in Cognitive Sciences, 15(9), 409–416. 10.1016/j.tics.2011.07.003 21855394 PMC3691066

[IMAG.a.1041-b28] Grasby, K. L., Jahanshad, N., Painter, J. N., Colodro-Conde, L., Bralten, J., Hibar, D. P., Lind, P. A., Pizzagalli, F., Ching, C. R. K., McMahon, M. A. B., Shatokhina, N., Zsembik, L. C. P., Thomopoulos, S. I., Zhu, A. H., Strike, L. T., Agartz, I., Alhusaini, S., Almeida, M. A. A., Alnæs, D.,…Enhancing NeuroImaging Genetics through Meta-Analysis Consortium (ENIGMA)—Genetics working group. (2020). The genetic architecture of the human cerebral cortex. Science, 367(6484), eaay6690. 10.1126/science.aay6690 32193296 PMC7295264

[IMAG.a.1041-b29] Grydeland, H., Walhovd, K. B., Tamnes, C. K., Westlye, L. T., & Fjell, A. M. (2013). Intracortical myelin links with performance variability across the human lifespan: Results from T1- and T2-weighted MRI myelin mapping and diffusion tensor imaging. The Journal of Neuroscience, 33(47), 18618–18630. 10.1523/JNEUROSCI.2811-13.2013 24259583 PMC6618798

[IMAG.a.1041-b30] Hardan, A. Y., Muddasani, S., Vemulapalli, M., Keshavan, M. S., & Minshew, N. J. (2006). An MRI study of increased cortical thickness in autism. The American Journal of Psychiatry, 163(7), 1290–1292. 10.1176/ajp.2006.163.7.1290 16816240 PMC1509104

[IMAG.a.1041-b31] Harris, A. D., Puts, N. A. J., Barker, P. B., & Edden, R. A. E. (2015). Spectral-editing measurements of GABA in the human brain with and without macromolecule suppression. Magnetic Resonance in Medicine, 74(6), 1523–1529. 10.1002/mrm.25549 25521836 PMC4470877

[IMAG.a.1041-b32] Harris, A. D., Saleh, M. G., & Edden, R. A. E. (2017). Edited 1 H magnetic resonance spectroscopy in vivo: Methods and metabolites. Magnetic Resonance in Medicine, 77(4), 1377–1389. 10.1002/mrm.26619 28150876 PMC5352552

[IMAG.a.1041-b33] Hasan, A., McIntosh, A. M., Droese, U.-A., Schneider-Axmann, T., Lawrie, S. M., Moorhead, T. W., Tepest, R., Maier, W., Falkai, P., & Wobrock, T. (2011). Prefrontal cortex gyrification index in twins: An MRI study. European Archives of Psychiatry and Clinical Neuroscience, 261(7), 459–465. 10.1007/s00406-011-0198-2 21336867 PMC3182317

[IMAG.a.1041-b34] Hill, J., Inder, T., Neil, J., Dierker, D., Harwell, J., & Van Essen, D. (2010). Similar patterns of cortical expansion during human development and evolution. Proceedings of the National Academy of Sciences of the United States of America, 107(29), 13135–13140. 10.1073/pnas.1001229107 20624964 PMC2919958

[IMAG.a.1041-b35] Huttenlocher, P. R. (1979). Synaptic density in human frontal cortex—Developmental changes and effects of aging. Brain Research, 163(2), 195–205. 10.1016/0006-8993(79)90349-4 427544

[IMAG.a.1041-b36] Huttenlocher, P. R., & Dabholkar, A. S. (1997). Regional differences in synaptogenesis in human cerebral cortex. The Journal of Comparative Neurology, 387(2), 167–178. 10.1002/(sici)1096-9861(19971020)387:2<167::aid-cne1>3.0.co;2-z 9336221

[IMAG.a.1041-b37] Huttenlocher, P. R., & de Courten, C. (1987). The development of synapses in striate cortex of man. Human Neurobiology, 6(1), 1–9. 10.1016/0304-3940(82)90379-2 3583840

[IMAG.a.1041-b38] Hyde, K. L., Samson, F., Evans, A. C., & Mottron, L. (2010). Neuroanatomical differences in brain areas implicated in perceptual and other core features of autism revealed by cortical thickness analysis and voxel-based morphometry. Human Brain Mapping, 31(4), 556–566. 10.1002/hbm.20887 19790171 PMC6870833

[IMAG.a.1041-b39] Jensen, J. E., deB. Frederick, B., & Renshaw, P. F. (2005). Grey and white matter GABA level differences in the human brain using two-dimensional, J-resolved spectroscopic imaging. NMR in Biomedicine, 18(8), 570–576. 10.1002/nbm.994 16273508

[IMAG.a.1041-b40] Jiang, L., Cao, X., Li, T., Tang, Y., Li, W., Wang, J., Chan, R. C., & Li, C. (2016). Cortical thickness changes correlate with cognition changes after cognitive training: Evidence from a Chinese community study. Frontiers in Aging Neuroscience, 8, 118. 10.3389/fnagi.2016.00118 27252649 PMC4877512

[IMAG.a.1041-b41] Jiang, X., Zhang, T., Zhang, S., Kendrick, K. M., & Liu, T. (2021). Fundamental functional differences between gyri and sulci: Implications for brain function, cognition, and behavior. Psychoradiology, 1(1), 23–41. 10.1093/psyrad/kkab002 38665307 PMC10939337

[IMAG.a.1041-b42] Kaas, J. H., & Stepniewska, I. (2016). Evolution of posterior parietal cortex and parietal-frontal networks for specific actions in primates. The Journal of Comparative Neurology, 524(3), 595–608. 10.1002/cne.23838 26101180 PMC4689678

[IMAG.a.1041-b43] Kaindl, A. M., Koppelstaetter, A., Nebrich, G., Stuwe, J., Sifringer, M., Zabel, C., Klose, J., & Ikonomidou, C. (2008). Brief alteration of NMDA or GABAA receptor-mediated neurotransmission has long term effects on the developing cerebral cortex. Molecular & Cellular Proteomics, 7(12), 2293–2310. 10.1074/mcp.M800030-MCP200 18587059

[IMAG.a.1041-b44] Kim, H.-J., Cho, M.-H., Shim, W. H., Kim, J. K., Jeon, E.-Y., Kim, D.-H., & Yoon, S.-Y. (2017). Deficient autophagy in microglia impairs synaptic pruning and causes social behavioral defects. Molecular Psychiatry, 22(11), 1576–1584. 10.1038/mp.2016.103 27400854 PMC5658669

[IMAG.a.1041-b45] Kinney, H. C., Brody, B. A., Kloman, A. S., & Gilles, F. H. (1988). Sequence of central nervous system myelination in human infancy. II. Patterns of myelination in autopsied infants. Journal of Neuropathology and Experimental Neurology, 47(3), 217–234. 10.1097/00005072-198805000-00003 3367155

[IMAG.a.1041-b46] Kirov, I. I., Fleysher, L., Fleysher, R., Patil, V., Liu, S., & Gonen, O. (2008). Age dependence of regional proton metabolites T2 relaxation times in the human brain at 3 T. Magnetic Resonance in Medicine, 60(4), 790–795. 10.1002/mrm.21715 18816831 PMC2631566

[IMAG.a.1041-b47] Klein, D., Rotarska-Jagiela, A., Genc, E., Sritharan, S., Mohr, H., Roux, F., Han, C. E., Kaiser, M., Singer, W., & Uhlhaas, P. J. (2014). Adolescent brain maturation and cortical folding: Evidence for reductions in gyrification. PLoS One, 9(1), e84914. 10.1371/journal.pone.0084914 24454765 PMC3893168

[IMAG.a.1041-b48] Kohli, J. S., Kinnear, M. K., Fong, C. H., Fishman, I., Carper, R. A., & Müller, R.-A. (2019). Local cortical gyrification is increased in children with autism spectrum disorders, but decreases rapidly in adolescents. Cerebral Cortex (New York, NY), 29(6), 2412–2423. 10.1093/cercor/bhy111 PMC6519693 29771286

[IMAG.a.1041-b49] Koolschijn, P. C. M. P., & Crone, E. A. (2013). Sex differences and structural brain maturation from childhood to early adulthood. Developmental Cognitive Neuroscience, 5, 106–118. 10.1016/j.dcn.2013.02.003 23500670 PMC6987760

[IMAG.a.1041-b50] la Fougère, C., Grant, S., Kostikov, A., Schirrmacher, R., Gravel, P., Schipper, H. M., Reader, A., Evans, A., & Thiel, A. (2011). Where in-vivo imaging meets cytoarchitectonics: The relationship between cortical thickness and neuronal density measured with high-resolution [18F]flumazenil-PET. NeuroImage, 56(3), 951–960. 10.1016/j.neuroimage.2010.11.015 21073964

[IMAG.a.1041-b51] Lebel, C., Walker, L., Leemans, A., Phillips, L., & Beaulieu, C. (2008). Microstructural maturation of the human brain from childhood to adulthood. NeuroImage, 40(3), 1044–1055. 10.1016/j.neuroimage.2007.12.053 18295509

[IMAG.a.1041-b52] Lin, A., Andronesi, O., Bogner, W., Choi, I., Coello, E., Cudalbu, C., Juchem, C., Kemp, G. J., Kreis, R., Krššák, M., Lee, P., Maudsley, A. A., Meyerspeer, M., Mlynarik, V., Near, J., Öz, G., Peek, A. L., Puts, N. A., Ratai, E., … Mullins, P. G. (2021). Minimum reporting standards for in vivo magnetic resonance spectroscopy (MRSinMRS): Experts’ consensus recommendations. NMR in Biomedicine, 34(5), e4484. 10.1002/nbm.4484 33559967 PMC8647919

[IMAG.a.1041-b53] Lohmann, C., & Kessels, H. W. (2013). The developmental stages of synaptic plasticity. The Journal of Physiology, 592(Pt 1), 13. 10.1113/jphysiol.2012.235119 24144877 PMC3903349

[IMAG.a.1041-b55] Luhmann, H. J., Fukuda, A., & Kilb, W. (2015). Control of cortical neuronal migration by glutamate and GABA. Frontiers in Cellular Neuroscience, 9, 4. 10.3389/fncel.2015.00004 25688185 PMC4311642

[IMAG.a.1041-b56] Madre, M., Canales-Rodríguez, E. J., Fuentes-Claramonte, P., Alonso-Lana, S., Salgado-Pineda, P., Guerrero-Pedraza, A., Moro, N., Bosque, C., Gomar, J. J., Ortíz-Gil, J., Goikolea, J. M., Bonnin, C. M., Vieta, E., Sarró, S., Maristany, T., McKenna, P. J., Salvador, R., & Pomarol-Clotet, E. (2020). Structural abnormality in schizophrenia versus bipolar disorder: A whole brain cortical thickness, surface area, volume and gyrification analyses. NeuroImage: Clinical, 25, 102131. 10.1016/j.nicl.2019.102131 31911343 PMC6948361

[IMAG.a.1041-b57] Menary, K., Collins, P. F., Porter, J. N., Muetzel, R., Olson, E. A., Kumar, V., Steinbach, M., Lim, K. O., & Luciana, M. (2013). Associations between cortical thickness and general intelligence in children, adolescents and young adults. Intelligence, 41(5), 597–606. 10.1016/j.intell.2013.07.010 24744452 PMC3985090

[IMAG.a.1041-b58] Mescher, M., Merkle, H., Kirsch, J., Garwood, M., & Gruetter, R. (1998). Simultaneous in vivo spectral editing and water suppression. NMR in Biomedicine, 11(6), 266–272. 10.1002/(SICI)1099-1492(199810)11:6<266::AID-NBM530>3.0.CO;2-J 9802468

[IMAG.a.1041-b59] Mikkelsen, M., Barker, P. B., Bhattacharyya, P. K., Brix, M. K., Buur, P. F., Cecil, K. M., Chan, K. L., Chen, D. Y.-T., Craven, A. R., Cuypers, K., Dacko, M., Duncan, N. W., Dydak, U., Edmondson, D. A., Ende, G., Ersland, L., Gao, F., Greenhouse, I., Harris, A. D., … Edden, R. A. E. (2017). Big GABA: Edited MR spectroscopy at 24 research sites. NeuroImage, 159, 32–45. 10.1016/j.neuroimage.2017.07.021 28716717 PMC5700835

[IMAG.a.1041-b60] Mikkelsen, M., He, J., Tommerdahl, M., Edden, R. A. E., Mostofsky, S. H., & Puts, N. A. J. (2020). Reproducibility of flutter-range vibrotactile detection and discrimination thresholds. Scientific Reports, 10, 6528. 10.1038/s41598-020-63208-z 32300187 PMC7162987

[IMAG.a.1041-b61] Mikkelsen, M., Tapper, S., Near, J., Mostofsky, S. H., Puts, N. A. J., & Edden, R. A. E. (2020). Correcting frequency and phase offsets in MRS data using robust spectral registration. NMR in Biomedicine, 33(10), e4368. 10.1002/nbm.4368 32656879 PMC9652614

[IMAG.a.1041-b62] Miller, B. L., Changl, L., Booth, R., Ernst, T., Cornford, M., Nikas, D., McBride, D., & Jenden, D. J. (1996). In vivo 1H MRS choline: Correlation with in vitro chemistry/histology. Life Sciences, 58(22), 1929–1935. 10.1016/0024-3205(96)00182-8 8637421

[IMAG.a.1041-b63] Miller, D. J., Duka, T., Stimpson, C. D., Schapiro, S. J., Baze, W. B., McArthur, M. J., Fobbs, A. J., Sousa, A. M. M., Sestan, N., Wildman, D. E., Lipovich, L., Kuzawa, C. W., Hof, P. R., & Sherwood, C. C. (2012). Prolonged myelination in human neocortical evolution. Proceedings of the National Academy of Sciences of the United States of America, 109(41), 16480–16485. 10.1073/pnas.1117943109 23012402 PMC3478650

[IMAG.a.1041-b64] Mlynárik, V., Gambarota, G., Frenkel, H., & Gruetter, R. (2006). Localized short-echo-time proton MR spectroscopy with full signal-intensity acquisition. Magnetic Resonance in Medicine, 56(5), 965–970. 10.1002/mrm.21043 16991116

[IMAG.a.1041-b65] Mlynárik, V., Gruber, S., & Moser, E. (2001). Proton T (1) and T (2) relaxation times of human brain metabolites at 3 Tesla. NMR in Biomedicine, 14(5), 325–331. 10.1002/nbm.713 11477653

[IMAG.a.1041-b66] Muftuler, L. T., Davis, E. P., Buss, C., Head, K., Hasso, A. N., & Sandman, C. A. (2011). Cortical and subcortical changes in typically developing preadolescent children. Brain Research, 1399, 15–24. 10.1016/j.brainres.2011.05.018 21640983 PMC3142577

[IMAG.a.1041-b67] Mullins, P. G., McGonigle, D. J., O’Gorman, R. L., Puts, N. A. J., Vidyasagar, R., Evans, C. J., Cardiff Symposium on MRS of GABA, & Edden, R. A. E. (2014). Current practice in the use of MEGA-PRESS spectroscopy for the detection of GABA. NeuroImage, 86, 43–52. 10.1016/j.neuroimage.2012.12.004 23246994 PMC3825742

[IMAG.a.1041-b68] Murali-Manohar, S., Zöllner, H. J., Hupfeld, K. E., Song, Y., Carter, E. E., Yedavalli, V., Hui, S. C. N., Simicic, D., Gudmundson, A. T., Simegn, G. L., Davies-Jenkins, C. W., Oeltzschner, G., Porges, E. C., & Edden, R. A. E. (2025). Age dependency of neurometabolite T1 relaxation times. Magnetic Resonance in Medicine, 94(2), 508–520. 10.1002/mrm.30507 40228052 PMC12670978

[IMAG.a.1041-b69] Narushima, M., Uchigashima, M., Yagasaki, Y., Harada, T., Nagumo, Y., Uesaka, N., Hashimoto, K., Aiba, A., Watanabe, M., Miyata, M., & Kano, M. (2016). The metabotropic glutamate receptor subtype 1 mediates experience-dependent maintenance of mature synaptic connectivity in the visual thalamus. Neuron, 91(5), 1097–1109. 10.1016/j.neuron.2016.07.035 27545713

[IMAG.a.1041-b70] Natu, V. S., Gomez, J., Barnett, M., Jeska, B., Kirilina, E., Jaeger, C., Zhen, Z., Cox, S., Weiner, K. S., Weiskopf, N., & Grill-Spector, K. (2019). Apparent thinning of human visual cortex during childhood is associated with myelination. Proceedings of the National Academy of Sciences, 116(41), 20750–20759. 10.1073/pnas.1904931116 PMC6789966 31548375

[IMAG.a.1041-b71] Near, J., Simpson, R., Cowen, P., & Jezzard, P. (2011). Efficient γ-aminobutyric acid editing at 3T without macromolecule contamination: MEGA-SPECIAL. NMR in Biomedicine, 24(10), 1277–1285. 10.1002/nbm.1688 21387450

[IMAG.a.1041-b72] Nie, J., Guo, L., Li, K., Wang, Y., Chen, G., Li, L., Chen, H., Deng, F., Jiang, X., Zhang, T., Huang, L., Faraco, C., Zhang, D., Guo, C., Yap, P.-T., Hu, X., Li, G., Lv, J., Yuan, Y., … Liu, T. (2012). Axonal fiber terminations concentrate on gyri. Cerebral Cortex (New York, N.Y.: 1991), 22(12), 2831–2839. 10.1093/cercor/bhr361 22190432 PMC3491768

[IMAG.a.1041-b73] Norbom, L. B., Ferschmann, L., Parker, N., Agartz, I., Andreassen, O. A., Paus, T., Westlye, L. T., & Tamnes, C. K. (2021). New insights into the dynamic development of the cerebral cortex in childhood and adolescence: Integrating macro- and microstructural MRI findings. Progress in Neurobiology, 204, 102109. 10.1016/j.pneurobio.2021.102109 34147583

[IMAG.a.1041-b74] Oeltzschner, G., Zöllner, H. J., Hui, S. C. N., Mikkelsen, M., Saleh, M. G., Tapper, S., & Edden, R. A. E. (2020). Osprey: Open-source processing, reconstruction & estimation of magnetic resonance spectroscopy data. Journal of Neuroscience Methods, 343, 108827. 10.1016/j.jneumeth.2020.108827 32603810 PMC7477913

[IMAG.a.1041-b75] Onwordi, E. C., Whitehurst, T., Mansur, A., Statton, B., Berry, A., Quinlan, M., O’Regan, D. P., Rogdaki, M., Marques, T. R., Rabiner, E. A., Gunn, R. N., Vernon, A. C., Natesan, S., & Howes, O. D. (2021). The relationship between synaptic density marker SV2A, glutamate and N-acetyl aspartate levels in healthy volunteers and schizophrenia: A multimodal PET and magnetic resonance spectroscopy brain imaging study. Translational Psychiatry, 11(1), 1–9. 10.1038/s41398-021-01515-3 34282130 PMC8290006

[IMAG.a.1041-b76] Ouyang, X., Pan, Y., Chen, X., Wu, G., Cheng, Y., Tan, W., Zhang, M., Deng, M., Liu, Z., & Palaniyappan, L. (2023). Cortical morphological heterogeneity of schizophrenia and its relationship with glutamatergic receptor variations. European Psychiatry: The Journal of the Association of European Psychiatrists, 66(1), e38. 10.1192/j.eurpsy.2023.2408 37158213 PMC10304990

[IMAG.a.1041-b77] Parker, N., Patel, Y., Jackowski, A. P., Pan, P. M., Salum, G. A., Pausova, Z., Paus, T., & Saguenay Youth Study and the IMAGEN Consortium. (2020). Assessment of neurobiological mechanisms of cortical thinning during childhood and adolescence and their implications for psychiatric disorders. JAMA Psychiatry, 77(11), 1127–1136. 10.1001/jamapsychiatry.2020.1495 32584945 PMC7301307

[IMAG.a.1041-b78] Paus, T., Keshavan, M., & Giedd, J. N. (2008). Why do many psychiatric disorders emerge during adolescence? Nature Reviews Neuroscience, 9(12), 947–957. 10.1038/nrn2513 19002191 PMC2762785

[IMAG.a.1041-b79] Peek, A. L., Rebbeck, T. J., Leaver, A. M., Foster, S. L., Refshauge, K. M., Puts, N. A., Oeltzschner, G., Andronesi, O. C., Barker, P. B., Bogner, W., Cecil, K. M., Choi, I.-Y., Deelchand, D. K., de Graaf, R. A., Dydak, U., Edden, R. A. E., Emir, U. E., Harris, A. D., Lin, A. P., … Wilson, M. (2023). A comprehensive guide to MEGA-PRESS for GABA measurement. Analytical Biochemistry, 669, 115113. 10.1016/j.ab.2023.115113 36958511 PMC10805000

[IMAG.a.1041-b80] Perdue, M. V., DeMayo, M. M., Bell, T. K., Boudes, E., Bagshawe, M., Harris, A. D., & Lebel, C. (2023). Changes in brain metabolite levels across childhood. NeuroImage, 274, 120087. 10.1016/j.neuroimage.2023.120087 37080345

[IMAG.a.1041-b81] Petanjek, Z., Judaš, M., Šimic, G., Rasin, M. R., Uylings, H. B. M., Rakic, P., & Kostovic, I. (2011). Extraordinary neoteny of synaptic spines in the human prefrontal cortex. Proceedings of the National Academy of Sciences of the United States of America, 108(32), 13281–13286. 10.1073/pnas.1105108108 21788513 PMC3156171

[IMAG.a.1041-b82] Pulli, E. P., Silver, E., Kumpulainen, V., Copeland, A., Merisaari, H., Saunavaara, J., Parkkola, R., Lähdesmäki, T., Saukko, E., Nolvi, S., Kataja, E.-L., Korja, R., Karlsson, L., Karlsson, H., & Tuulari, J. J. (2022). Feasibility of FreeSurfer processing for T1-weighted brain images of 5-year-olds: Semiautomated protocol of FinnBrain neuroimaging lab. Frontiers in Neuroscience, 16, 874062. 10.3389/fnins.2022.874062 35585923 PMC9108497

[IMAG.a.1041-b83] Puts, N. A. J., Barker, P. B., & Edden, R. A. E. (2013). Measuring the longitudinal relaxation time of GABA in vivo at 3 Tesla. Journal of Magnetic Resonance Imaging: JMRI, 37(4), 999–1003. 10.1002/jmri.23817 23001644 PMC3531569

[IMAG.a.1041-b84] Puts, N. A. J., & Edden, R. A. E. (2012). In vivo magnetic resonance spectroscopy of GABA: A methodological review. Progress in Nuclear Magnetic Resonance Spectroscopy, 60, 29–41. 10.1016/j.pnmrs.2011.06.001 22293397 PMC3383792

[IMAG.a.1041-b85] Puts, N. A. J., Wodka, E. L., Harris, A. D., Crocetti, D., Tommerdahl, M., Mostofsky, S. H., & Edden, R. A. E. (2017). Reduced GABA and altered somatosensory function in children with autism spectrum disorder. Autism Research, 10(4), 608–619. 10.1002/aur.1691 27611990 PMC5344784

[IMAG.a.1041-b86] Puts, N., Mikkelsen, M., Mostofsky, S. H., & Edden, R. (2017). 2017 International meeting for autism research: Movement during MR scanning in children with autism spectrum disorder. https://insar.confex.com/insar/2017/webprogram/Paper23610.html

[IMAG.a.1041-b87] Rae, C. D. (2014). A guide to the metabolic pathways and function of metabolites observed in human brain 1H magnetic resonance spectra. Neurochemical Research, 39(1), 1–36. 10.1007/s11064-013-1199-5 24258018

[IMAG.a.1041-b88] Reyngoudt, H., Claeys, T., Vlerick, L., Verleden, S., Acou, M., Deblaere, K., De Deene, Y., Audenaert, K., Goethals, I., & Achten, E. (2012). Age-related differences in metabolites in the posterior cingulate cortex and hippocampus of normal ageing brain: A 1H-MRS study. European Journal of Radiology, 81(3), e223–e231. 10.1016/j.ejrad.2011.01.106 21345628

[IMAG.a.1041-b89] Ronan, L., Voets, N., Rua, C., Alexander-Bloch, A., Hough, M., Mackay, C., Crow, T. J., James, A., Giedd, J. N., & Fletcher, P. C. (2014). Differential tangential expansion as a mechanism for cortical gyrification. Cerebral Cortex (New York, NY), 24(8), 2219–2228. 10.1093/cercor/bht082 PMC4089386 23542881

[IMAG.a.1041-b90] Sailasuta, N., Ernst, T., & Chang, L. (2008). Regional variations and the effects of age and gender on glutamate concentrations in the human brain. Magnetic Resonance Imaging, 26(5), 667–675. 10.1016/j.mri.2007.06.007 17692491 PMC2712610

[IMAG.a.1041-b91] Saleh, M. G., Edden, R. A. E., Chang, L., & Ernst, T. (2020). Motion correction in magnetic resonance spectroscopy. Magnetic Resonance in Medicine, 84(5), 2312–2326. 10.1002/mrm.28287 32301174 PMC8386494

[IMAG.a.1041-b92] Schaer, M., Cuadra, M. B., Tamarit, L., Lazeyras, F., Eliez, S., & Thiran, J.-P. (2008). A surface-based approach to quantify local cortical gyrification. IEEE Transactions on Medical Imaging, 27(2), 161–170. 10.1109/TMI.2007.903576 18334438

[IMAG.a.1041-b93] Selemon, L. D. (2013). A role for synaptic plasticity in the adolescent development of executive function. Translational Psychiatry, 3(3), e238. 10.1038/tp.2013.7 23462989 PMC3625918

[IMAG.a.1041-b94] Shin, J., French, L., Xu, T., Leonard, G., Perron, M., Pike, G. B., Richer, L., Veillette, S., Pausova, Z., & Paus, T. (2018). Cell-specific gene-expression profiles and cortical thickness in the human brain. Cerebral Cortex (New York, N.Y.: 1991), 28(9), 3267–3277. 10.1093/cercor/bhx197 28968835 PMC13017599

[IMAG.a.1041-b95] Simpson, R., Devenyi, G. A., Jezzard, P., Hennessy, T. J., & Near, J. (2017). Advanced processing and simulation of MRS data using the FID appliance (FID-A)-An open source, MATLAB-based toolkit. Magnetic Resonance in Medicine, 77(1), 23–33. 10.1002/mrm.26091 26715192

[IMAG.a.1041-b96] Sowell, E. R., Thompson, P. M., Leonard, C. M., Welcome, S. E., Kan, E., & Toga, A. W. (2004). Longitudinal mapping of cortical thickness and brain growth in normal children. The Journal of Neuroscience: The Official Journal of the Society for Neuroscience, 24(38), 8223–8231. 10.1523/JNEUROSCI.1798-04.2004 15385605 PMC6729679

[IMAG.a.1041-b97] Surdin, T., Preissing, B., Rohr, L., Grömmke, M., Böke, H., Barcik, M., Azimi, Z., Jancke, D., Herlitze, S., Mark, M. D., & Siveke, I. (2023). Optogenetic activation of mGluR1 signaling in the cerebellum induces synaptic plasticity. iScience, 26(1), 105828. 10.1016/j.isci.2022.105828 36632066 PMC9826949

[IMAG.a.1041-b99] Thomson, A. R., Hwa, H., Pasanta, D., Hopwood, B., Powell, H. J., Lawrence, R., Tabuenca, Z. G., Arichi, T., Edden, R. A. E., Chai, X., & Puts, N. A. (2024). The developmental trajectory of 1H-MRS brain metabolites from childhood to adulthood. Cerebral Cortex (New York, N.Y.: 1991), 34(3), bhae046. 10.1093/cercor/bhae046 38430105 PMC10908220

[IMAG.a.1041-b100] Thomson, A. R., Pasanta, D., Arichi, T., & Puts, N. A. (2024). Neurometabolite differences in Autism as assessed with magnetic resonance spectroscopy: A systematic review and meta-analysis. Neuroscience & Biobehavioral Reviews, 162, 105728. 10.1016/j.neubiorev.2024.105728 38796123 PMC11602446

[IMAG.a.1041-b101] Tran, H., Le, L., Singh, B. N., Kramer, J., & Steward, R. (2024). Tet controls axon guidance in early brain development through glutamatergic signaling. iScience, 27(5), 109634. 10.1016/j.isci.2024.109634 38655199 PMC11035372

[IMAG.a.1041-b102] van der Meer, D., Frei, O., Kaufmann, T., Chen, C.-H., Thompson, W. K., O’Connell, K. S., Monereo Sánchez, J., Linden, D. E. J., Westlye, L. T., Dale, A. M., & Andreassen, O. A. (2020). Quantifying the polygenic architecture of the human cerebral cortex: Extensive genetic overlap between cortical thickness and surface area. Cerebral Cortex, 30(10), 5597–5603. 10.1093/cercor/bhaa146 32483632 PMC7472200

[IMAG.a.1041-b103] Veenstra-VanderWeele, J., Cook, E. H., King, B. H., Zarevics, P., Cherubini, M., Walton-Bowen, K., Bear, M. F., Wang, P. P., & Carpenter, R. L. (2017). Arbaclofen in children and adolescents with autism spectrum disorder: A randomized, controlled, phase 2 trial. Neuropsychopharmacology, 42(7), 1390–1398. 10.1038/npp.2016.237 27748740 PMC5436109

[IMAG.a.1041-b104] Vidal-Pineiro, D., Parker, N., Shin, J., French, L., Grydeland, H., Jackowski, A. P., Mowinckel, A. M., Patel, Y., Pausova, Z., Salum, G., Sørensen, Ø., Walhovd, K. B., Paus, T., & Fjell, A. M. (2020). Cellular correlates of cortical thinning throughout the lifespan. Scientific Reports, 10(1), 21803. 10.1038/s41598-020-78471-3 33311571 PMC7732849

[IMAG.a.1041-b105] Voevodskaya, O., Simmons, A., Nordenskjöld, R., Kullberg, J., Ahlström, H., Lind, L., Wahlund, L.-O., Larsson, E.-M., & Westman, E. (2014). The effects of intracranial volume adjustment approaches on multiple regional MRI volumes in healthy aging and Alzheimer’s disease. Frontiers in Aging Neuroscience, 6, 264. 10.3389/fnagi.2014.00264 25339897 PMC4188138

[IMAG.a.1041-b106] Wallimann, T., Wyss, M., Brdiczka, D., Nicolay, K., & Eppenberger, H. M. (1992). Intracellular compartmentation, structure and function of creatine kinase isoenzymes in tissues with high and fluctuating energy demands: The ‘phosphocreatine circuit’ for cellular energy homeostasis. Biochemical Journal, 281(Pt 1), 21–40. 10.1042/bj2810021 1731757 PMC1130636

[IMAG.a.1041-b107] White, T., Su, S., Schmidt, M., Kao, C.-Y., & Sapiro, G. (2010). The development of gyrification in childhood and adolescence. Brain and Cognition, 72(1), 36. 10.1016/j.bandc.2009.10.009 19942335 PMC2815169

[IMAG.a.1041-b109] You, W., Li, Q., Chen, L., He, N., Li, Y., Long, F., Wang, Y., Chen, Y., McNamara, R. K., Sweeney, J. A., DelBello, M. P., Gong, Q., & Li, F. (2024). Common and distinct cortical thickness alterations in youth with autism spectrum disorder and attention-deficit/hyperactivity disorder. BMC Medicine, 22(1), 92. 10.1186/s12916-024-03313-2 38433204 PMC10910790

[IMAG.a.1041-b110] Zecevic, N., Bourgeois, J. P., & Rakic, P. (1989). Changes in synaptic density in motor cortex of rhesus monkey during fetal and postnatal life. Brain Research. Developmental Brain Research, 50(1), 11–32. 10.1016/0165-3806(89)90124-7 2582602

[IMAG.a.1041-b111] Zhang, Y., & Shen, J. (2015). Regional and tissue-specific differences in brain glutamate concentration measured by in vivo single voxel MRS. Journal of Neuroscience Methods, 239, 94–99. 10.1016/j.jneumeth.2014.09.021 25261738 PMC4314957

[IMAG.a.1041-b112] Zhou, Z., Wei, D., Liu, W., Chen, H., Qin, S., Xu, P., Zuo, X.-N., Luo, Y.-J., & Qiu, J. (2023). Gene transcriptional expression of cortical thinning during childhood and adolescence. Human Brain Mapping, 44(10), 4040–4051. 10.1002/hbm.26328 37146003 PMC10258537

