# Supplementary material for: Characterising the association between posterior parietal metabolite levels and cortical macrostructure in a cohort spanning childhood to adulthood
Source: Imaging Neurosci (Camb). 2025 Dec 3;3:IMAG.a.1041. doi: 10.1162/IMAG.a.1041 (PMC13277667; doi:10.1162/IMAG.a.1041)
Supplement: Supplementary Material [file IMAG.a.1041_supp.pdf]

Supplementary Materials

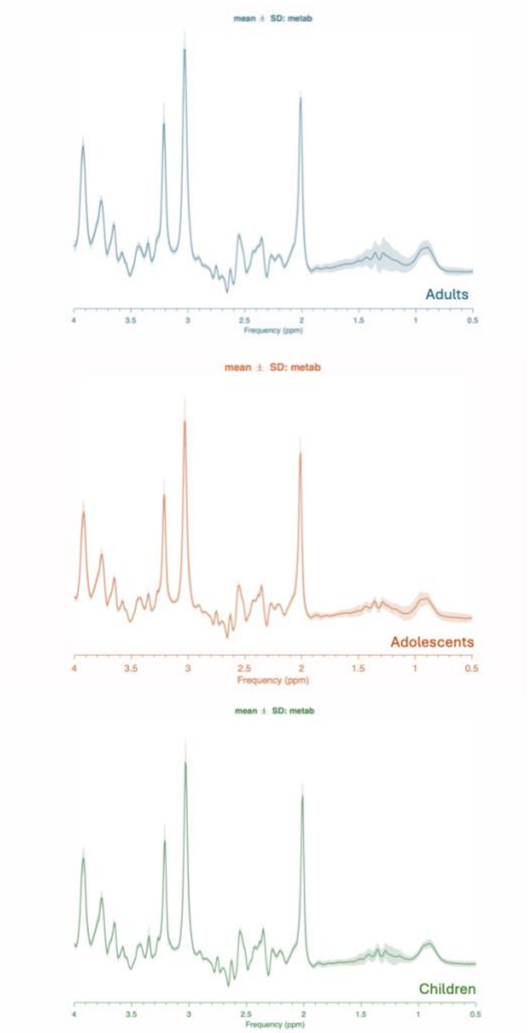

Supplementary Figure 1. Mean MEGA-PRESS SUM spectra per age group, showing model fit and median residuals (error bars).

Supplementary Table 1. MRS data quality metrics per age group. Medians (interquartile range (IQR)) are shown. Quality metrics include signal to noise ratio (SNR), full-width half maximum (FWHM; Hertz (Hz)), fit residuals (edit-OFF and difference spectra) and frequency shift (Hz). Gray matter (GM), white matter (WM) and cerebral spinal fluid (CSF) voxel fractions are also shown.

QM	***Child***	***Adolescent***	***Adult***	***All***
SNR	217.78 (27.53)	195.98 (34.99)	169.42 (30.57)	191.73 (53.62)
FWHM (Hz)	4.26 (0.34)	4.56 (0.49)	5.05 (0.56)	4.63 (0.74)
Fit residuals (difference)	3.56 (1.01)	2.92 (1.32)	2.57 (0.80)	2.98 (1.06)
Fit residuals (OFF)	16.10 (4.29)	11.70 (5.24)	8.49 (2.88)	11.73 (6.83)
Frequency shift (Hz)	−2.42 (0.44)	−2.38 (0.86)	−2.39 (0.82)	−2.39 (0.78)
GM fraction	0.70 (0.061)	0.65 (0.040)	0.61 (0.041)	0.64 (0.073)
WM fraction	0.24 (0.052)	0.27 (0.039)	0.30 (0.046)	0.27 (0.059)
CSF fraction	0.059 (0.029)	0.072 (0.031)	0.085 (0.040)	0.073 (0.035)

Supplementary Table 2. Tissue-specific metabolite and water T1 and T2 relaxation times used for tissue-correction of water-scaled metabolite concentrations according to (Gasparovic et al., 2006). T1 values for NAA, Glu, Cr, Cho, mI were obtained from from (Mlynárik et al., 2001). T1 for GABA was obtained from (Puts et al., 2013). Water relaxation values were obtained from (Lu et al., 2005).

Metabolite	***T1_GM***	***T1_WM***	***T2_GM***	***T2_WM***

ASC	1340	1190	(125+105)/2	172
ASP	1340	1190	(111+190)/2	148
CR	1460	1240	(148+144)/2	166
GABA	1310	1310	(102+75)/2	(102+75)/2
GLC	1340	1190	(117+88)/2	155
GLN	1340	1190	(122+99)/2	168
GLU	1270	1170	(135+122)/2	124
GLY	1340	1190	(102+81)/2	152
GPC	1300	1080	(274+222)/2	218
GSH	1340	1190	(100+77)/2	145
LAC	1340	1190	(110+99)/2	159
mI	1230	1010	(244+229)/2	161
NAA	1470	1350	(253+263)/2	343
NAAG	1340	1190	(128+107)/2	185
PCH	1300	1080	(274+221)/2	213
PCR	1460	1240	(148+144)/2	166
PE	1340	1190	(119+86)/2	158
SCY	1340	1190	(125+107)/2	170
TAU	1340	1190	(123+102)/2	(123+102)/2
tNAA	(1470+1340)/2	(1350+1190)/2	(253+263+128+107)/4	(343+185)/2];
tCr	(1460+1460)/2	(1240+1240)/2	(148+144+148+144)/4	(166+166)/2
tCho	(1300+1080)/2	(1080+1080)/2	(274+222+274+221)/4	(218+213)/2
Glx	(1340+1270)/2	(1190+1170)/2	(122+99+135+122)/4	(168+124)/2
water	1331	832	110	79.2

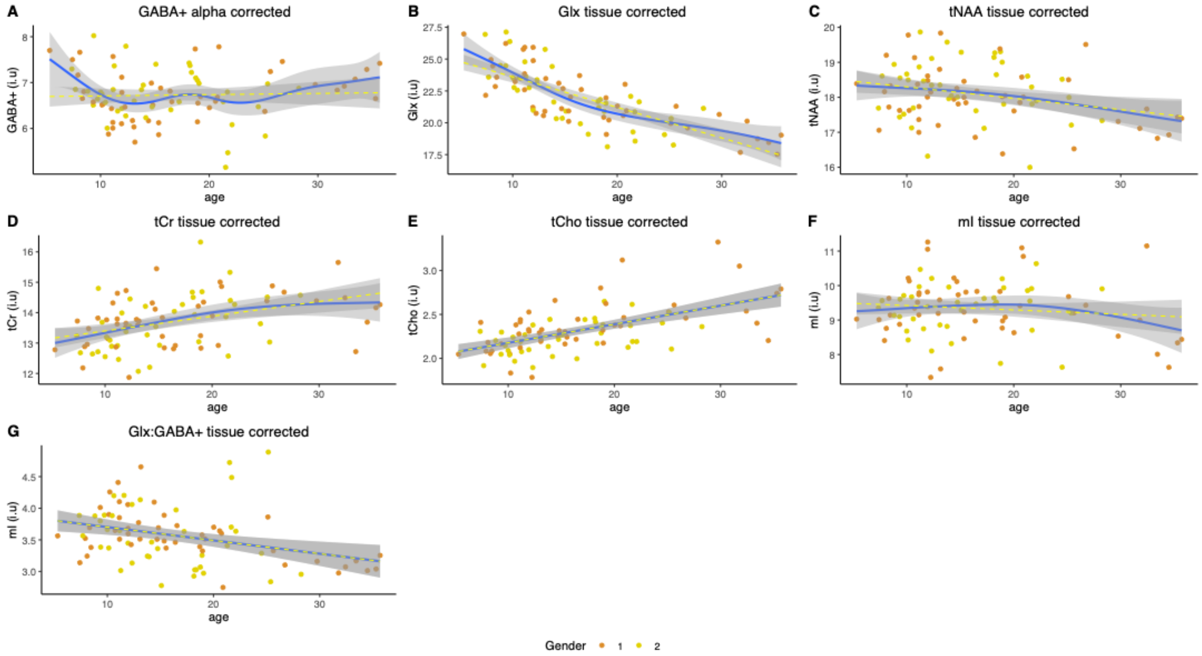

Supplementary Figure 2. Linear (yellow) and GAM modelling (blue) of estimated metabolite concentrations (i.u) over the lifespan. (G) Also shown is the relationship between estimated Glx/GABA+ ratio and age. Orange point = Male, Yellow point = Female. Note significant non-linear effects of age were identified in GAMS for GABA+ (edf = 4.48, $P < 0.05$), tCr (edf = 1.43, $P < 0.05$) and Glx (edf = 1.59, $P < 0.001$). Linear age effects were significant and negative for estimated concentrations of Glx (beta = −0.14, $P < 0.05$), and significant and positive for estimated concentrations of tCr (beta = 0.043, $P < 0.05$) and tCho (beta = 0.020, p = 0). Sex effects were observed for tCho. Male and female trajectories are similar, with gradual increases in tCho concentrations across the lifespan, although this trend was more non-linear in males compared to females (edf_males = 2.14, $P < 0.01$; edf_females = 1, $P > 0.05$).

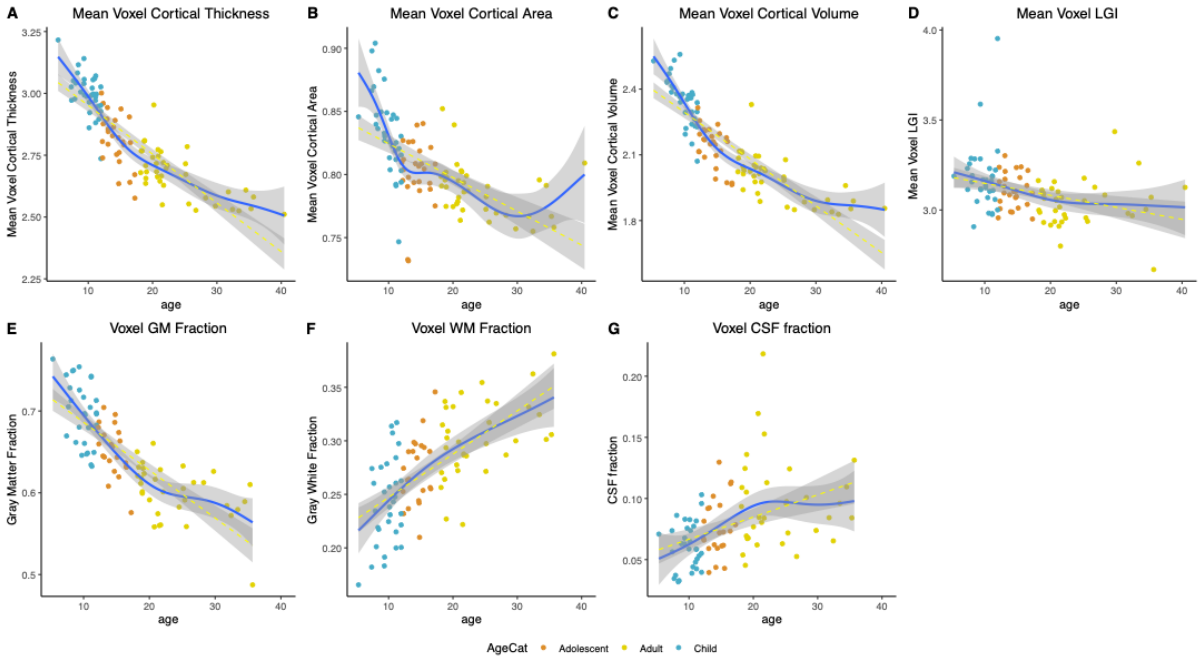

Supplementary Figure 3. PPC voxel cortical surface-based metrics plotted against participant age. (A) Mean PPC voxel cortical thickness (B) Mean PPC voxel cortical area (C) Mean PPC voxel cortical volume (D) Mean PPC voxel local gyrification index (LGI) (E) PPC voxel GM fraction (F) PPC voxel WM fraction (G) PPC voxel CSF fraction. Linear (yellow-hashed line) and non-linear GAM models (blue line) are shown. Blue point = child, orange point = adolescent, yellow point = adult.

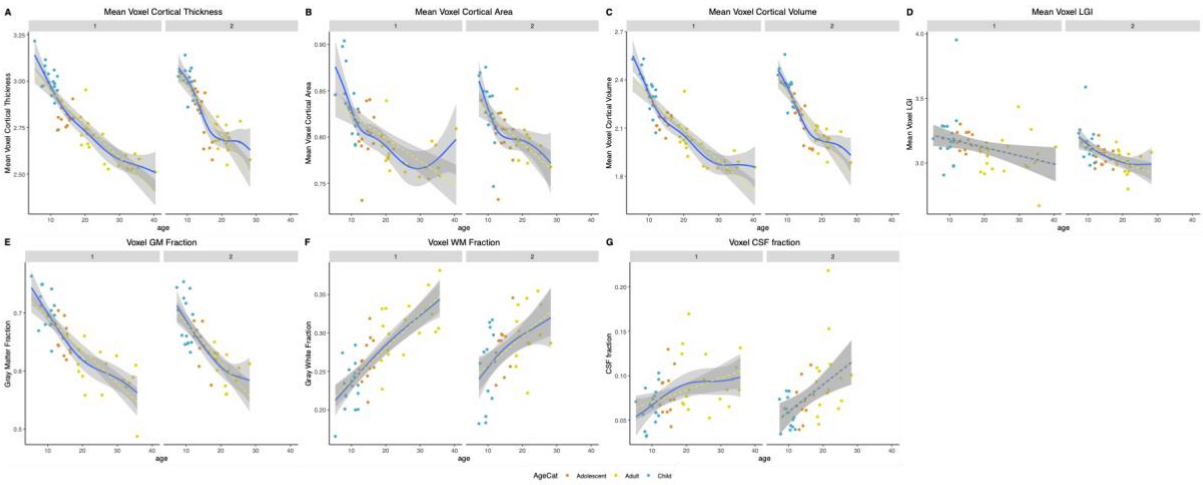

Supplementary Figure 4. PPC voxel cortical surface-based metrics plotted against participant age plotted by sex, 1 = Male, 2 = Female. (A) Mean PPC voxel cortical thickness (B) Mean PPC voxel cortical area (C) Mean PPC voxel cortical volume (D) Mean voxel local gyrification index (LGI) (E) PPC voxel GM fraction (F) PPC voxel WM fraction (G) PPC voxel CSF fraction. Linear (yellow-hashed line) and non-linear GAM models (blue line) are shown. Blue point = child, orange point = adolescent, yellow point = adult.

Supplementary Table 3. Results from linear regression of voxel structural metrics with age, IQ, eTIV and sex. Estimate = linear regression beta predictor coefficient. * Indicates significant p values at $p < 0.05$. R^2 = adjusted R^2 obtained from linear modelling.

Metric	*Term*	*Estimate*	*P value*	*R squared*	*Significance*
Mean Voxel Cortical Thickness	Intercept	3.286	0	0.743	*

	Age	-0.025	o	0.743	*
	Sex - Male	-0.101	0.045	0.743	*
	IQ	-0.001	0.248	0.743	
	eTIV	0.00	0.642	0.743	
	Age*Sex	0.007	0.016	0.743	*
Mean Voxel Cortical Area	Intercept	0.944	0	0.43	*
	Age	-0.003	0	0.43	*
	Sex - Male	-0.005	0.745	0.43	
	IQ	0	0.741	0.43	
	eTIV	0	0.002	0.43	*
	Age*Sex	0.001	0.345	0.43	
Mean Voxel Cortical Volume	Intercept	2.694	0	0.746	*
	Age	-0.028	0	0.746	*
	Sex - Male	-0.12	0.027	0.746	*
	IQ	-0.001	0.398	0.746	
	eTIV	0	0.861	0.746	
	Age*Sex	0.009	0.009	0.746	*
Mean Voxel Cortical LGI	Intercept	2.776	0	0.272	*
	Age	-0.01	0.015	0.272	*
	Sex - Male	0.003	0.965	0.272	
	IQ	0	0.887	0.272	
	eTIV	0	0.001	0.272	*
	Age*Sex	0.002	0.632	0.272	
Voxel GM Fraction	Intercept	0.724	0	0.657	*
	Age	-0.007	0	0.657	*
	Sex - Male	-0.001	0.971	0.657	
	IQ	0	0.317	0.657	
	eTIV	0	0.085	0.657	
	Age*Sex	0.001	0.538	0.657	
Voxel WM Fraction	Intercept	0.304	0	0.490	*
	Age	0.003	0.002	0.490	*
	Sex - Male	-0.029	0.138	0.490	
	IQ	0	0.799	0.490	
	eTIV	0	0.041	0.490	*
	Age*Sex	0.001	0.302	0.490	
Voxel CSF Fraction	Intercept	-0.027	0.530	0.244	
	Age	0.003	0.001	0.244	*
	Sex - Male	0.030	0.083	0.244	
	IQ	0	0.152	0.244	
	eTIV	0	0.708	0.244	
	Age*Sex	-0.002	0.061	0.244	

Supplementary Table 4. Results from GAM regression of PPC voxel mean metrics predicted by smooth functions of age, IQ, eTIV and sex as a categorical predictor. Estimate refers to beta coefficients for parametric terms, and the edf value for non-parametric smooth terms (complexity of the smooth function). R^2 = estimated R squared values for each GAM. * Indicates significant edf or beta coefficients at $p < 0.05$.

Model	*Term*	*Estimate*	*P value*	*R squared*	*Significance*
Mean Voxel Cortical Thickness	s(age)	4.17	0	0.792	*
	s(IQ)	1.00	0.464	0.792	
	s(eTIV)	1.22	0.409	0.792	
Mean Voxel Cortical Area	s(age)	1.85	0	0.401	*
	s(mean_white_surf_area)	4.43	0.004	0.401	*
	s(IQ)	1.00	0.501	0.401	
Mean Voxel Cortical Volume	s(age)	4.75	0	0.823	*
	s(eTIV)	1.11	0.679	0.823	
	s(IQ)	1.00	0.653	0.823	
Mean Voxel Cortical LGI	s(age)	1.87	0	0.328	*
	s(IQ)	2.85	0.297	0.328	
	s(eTIV)	2.49	0.001	0.328	*
Voxel GM Fraction	s(age)	3.22	0	0.701	*
	s(eTIV)	2.18	0.075	0.701	
	s(IQ)	1.00	0.783	0.701	
Voxel WM Fraction	s(age)	1.86	0	0.467	*
	s(eTIV)	1.00	0.056	0.467	
	s(IQ)	1.00	0.626	0.467	
Voxel CSF Fraction	s(age)	2.65	0	0.237	*
	s(eTIV)	1.74	0.576	0.237	
	s(IQ)	1.00	0.259	0.237	

Supplementary Table 5. Results from linear regression of mean voxel cortical thickness predicted by metabolite concentrations, age, IQ, eTIV, frequency shift and sex. Estimate = linear regression predictor beta coefficient. * Indicates significant p values at; '****' $p < 0$ '***' $p < 0.001$ '**' $p < 0.01$ '*' $p < 0.05$. R^2 = adjusted R^2 obtained from linear modelling.

Metabolite	***Variable***	***Estimate***	***p_value***	***R_squared***	***FDR_p_value***	***Significance_FDR***
Voxel Mean Cortical Thickness - GABAplus_diff1_AlphaCorrWaterScaled	(Intercept)	2.918	0	0.766	0	***
	GABAplus_diff1_AlphaCorrWaterScaled	0.045	0.024	0.766	0.074	.
	age	-0.02	0	0.766	0	***
	IQ	-0.001	0.208	0.766	0.557	

	Sex - Female	-0.009	0.698	0.766	0.843	
	eTIV	0	0.658	0.766	0.843	
	freqShift	-0.007	0.67	0.766	0.843	
Voxel Mean Cortical Thickness - GABAplus_diff1_TissCorrWaterScaled	(Intercept)	2.895	0	0.765	0	***
	GABAplus_diff1_TissCorrWaterScaled	0.059	0.027	0.765	0.077	
	age	-0.02	0	0.765	0	***
	IQ	-0.001	0.231	0.765	0.557	
	Sex - Female	-0.008	0.726	0.765	0.843	
	eTIV	0	0.708	0.765	0.843	
	freqShift	-0.007	0.688	0.765	0.843	
Voxel Mean Cortical Thickness - GABAplus_diff1_CSFWaterScaled	(Intercept)	2.875	0	0.767	0	***
	GABAplus_diff1_CSFWaterScaled	0.067	0.02	0.767	0.064	
	age	-0.02	0	0.767	0	***
	IQ	-0.001	0.241	0.767	0.557	
	Sex - Female	-0.008	0.711	0.767	0.843	
	eTIV	0	0.713	0.767	0.843	
	freqShift	-0.008	0.649	0.767	0.843	
Voxel Mean Cortical Thickness - Glx_A_TissCorrWaterScaled	(Intercept)	2.699	0	0.777	0	***
	Glx_A_TissCorrWaterScaled	0.019	0.003	0.777	0.011	*
	age	-0.016	0	0.777	0	***
	IQ	-0.001	0.39	0.777	0.735	
	Sex - Female	-0.005	0.816	0.777	0.843	
	eTIV	0	0.768	0.777	0.843	
	freqShift	-0.003	0.831	0.777	0.848	
Voxel Mean Cortical Thickness - Glx_A_CSFWaterScaled	(Intercept)	2.663	0	0.78	0	***
	Glx_A_CSFWaterScaled	0.024	0.002	0.78	0.007	**
	age	-0.015	0	0.78	0	***
	IQ	-0.001	0.42	0.78	0.776	
	Sex - Female	-0.006	0.802	0.78	0.843	
	eTIV	0	0.771	0.78	0.843	
	freqShift	-0.004	0.783	0.78	0.843	
Voxel Mean Cortical Thickness - tNAA_A_TissCorrWaterScaled	(Intercept)	3.362	0	0.75	0	***
	tNAA_A_TissCorrWaterScaled	-0.005	0.682	0.75	0.843	
	age	-0.02	0	0.75	0	***
	IQ	-0.001	0.299	0.75	0.598	
	Sex - Female	-0.006	0.817	0.75	0.843	
	eTIV	0	0.727	0.75	0.843	
	freqShift	0.011	0.534	0.75	0.843	
Voxel Mean Cortical Thickness - tNAA_A_CSFWaterScaled	(Intercept)	3.317	0	0.749	0	***
	tNAA_A_CSFWaterScaled	-0.003	0.813	0.749	0.843	
	age	-0.02	0	0.749	0	***
	IQ	-0.001	0.279	0.749	0.590	
	Sex - Female	-0.006	0.789	0.749	0.843	
	eTIV	0	0.73	0.749	0.843	
	freqShift	0.01	0.572	0.749	0.843	
Voxel Mean Cortical Thickness - tCr_A_TissCorrWaterScaled	(Intercept)	3.69	0	0.766	0	***
	tCr_A_TissCorrWaterScaled	-0.029	0.026	0.766	0.077	
	age	-0.019	0	0.766	0	***
	IQ	-0.001	0.284	0.766	0.59	
	Sex - Female	-0.009	0.683	0.766	0.843	
	eTIV	0	0.952	0.766	0.962	
	freqShift	0.02	0.233	0.766	0.557	
Voxel Mean Cortical Thickness - tCr_A_CSFWaterScaled	(Intercept)	3.685	0	0.764	0	***
	tCr_A_CSFWaterScaled	-0.032	0.033	0.764	0.093	
	age	-0.019	0	0.764	0	***
	IQ	-0.001	0.265	0.764	0.59	
	Sex - Female	-0.009	0.698	0.764	0.843	
	eTIV	0	0.967	0.764	0.967	
	freqShift	0.02	0.236	0.764	0.557	
Voxel Mean Cortical Thickness - tCho_A_TissCorrWaterScaled	(Intercept)	3.317	0	0.751	0	***
	tCho_A_TissCorrWaterScaled	-0.032	0.525	0.751	0.843	
	age	-0.019	0	0.751	0	***
	IQ	-0.001	0.244	0.751	0.557	
	Sex - Female	-0.009	0.696	0.751	0.843	
	eTIV	0	0.663	0.751	0.843	
	freqShift	0.011	0.505	0.751	0.843	
Voxel Mean Cortical Thickness - tCho_A_CSFWaterScaled	(Intercept)	3.31	0	0.75	0	***
	tCho_A_CSFWaterScaled	-0.025	0.574	0.75	0.843	
	age	-0.019	0	0.75	0	***
	IQ	-0.001	0.243	0.75	0.557	
	Sex - Female	-0.009	0.703	0.75	0.843	
	eTIV	0	0.67	0.75	0.843	
	freqShift	0.011	0.516	0.75	0.843	
Voxel Mean Cortical Thickness - mI_A_TissCorrWaterScaled	(Intercept)	3.382	0	0.753	0	***
	mI_A_TissCorrWaterScaled	-0.013	0.31	0.753	0.607	
	age	-0.02	0	0.753	0	***
	IQ	-0.001	0.289	0.753	0.590	
	Sex - Female	-0.009	0.717	0.753	0.843	
	eTIV	0	0.767	0.753	0.843	
	freqShift	0.009	0.576	0.753	0.843	
Voxel Mean Cortical Thickness - mI_A_CSFWaterScaled	(Intercept)	3.372	0	0.752	0	***
	mI_A_CSFWaterScaled	-0.012	0.356	0.752	0.685	
	age	-0.02	0	0.752	0	***
	IQ	-0.001	0.28	0.752	0.590	
	Sex - Female	-0.008	0.723	0.752	0.843	
	eTIV	0	0.761	0.752	0.843	
	freqShift	0.009	0.576	0.752	0.843	
Voxel Mean Cortical Thickness – GlxGABAalpharatio (tissue-corrected)	(Intercept)	3.322	0	0.751	0	***

GlxGABAalpharatio	-0.019	0.461	0.751	0.836	
age	-0.02	0	0.751	0	***
IQ	-0.001	0.239	0.751	0.557	
Sex - Female	-0.009	0.717	0.751	0.843	
eTIV	0	0.72	0.751	0.843	
freqShift	0.005	0.78	0.751	0.843	

Supplementary Table 6. Results from GAM regression of PPC mean cortical thickness predicted by smooth functions of age, and linear functionals of metabolite concentration, IQ, eTIV and sex as a categorical predictor. Estimate refers to beta coefficients for parametric terms, and the edf value for non-parametric smooth terms (complexity of the smooth function). R2 = estimated R squared values for each GAM. * Indicates significant edf or beta coefficients at; '****' p < 0 '***' p < 0.001 '**' p < 0.01 '*' p < 0.05.

Metabolite	*Variable*	*TermType*	*Estimate*	*p_value*	*Adjusted_ R2*	*FDR_p_value*	*Significance_ FDR*
Voxel Mean Cortical thickness - GABAplus_diff1_AlphaCorrWater Scaled	(Intercept)	Parametric	2.632	0	0.809	0	***
	GABAplus_diff1_AlphaCorrWat erScaled	Parametric	0.026	0.158	0.809	0.468	
	IQ	Parametric	0	0.596	0.809	0.852	
	eTIV	Parametric	0	0.592	0.809	0.852	
	Sex - Female	Parametric	0.01	0.613	0.809	0.852	
	freqShift	Parametric	-0.006	0.702	0.809	0.852	
	s(age)	Smooth	3.313	0	0.809	0	***
Voxel Mean Cortical thickness - GABAplus_diff1_TissCorrWaterSc aled	(Intercept)	Parametric	2.63	0	0.808	0	***
	GABAplus_diff1_TissCorrWater Scaled	Parametric	0.032	0.183	0.808	0.526	
	IQ	Parametric	0	0.62	0.808	0.852	
	eTIV	Parametric	0	0.615	0.808	0.852	
	Sex - Female	Parametric	0.011	0.595	0.808	0.852	
	freqShift	Parametric	-0.005	0.728	0.808	0.87	
	s(age)	Smooth	3.321	0	0.808	0	***
	(Intercept)	Parametric	2.609	0	0.81	0	***
Voxel Mean Cortical thickness - GABAplus_diff1_CSFWaterScaled	GABAplus_diff1_CSFWaterScal ed	Parametric	0.038	0.142	0.81	0.448	
	IQ	Parametric	0	0.626	0.81	0.852	
	eTIV	Parametric	0	0.614	0.81	0.852	
	Sex - Female	Parametric	0.01	0.607	0.81	0.852	
	freqShift	Parametric	-0.006	0.683	0.81	0.852	
	s(age)	Smooth	3.333	0	0.81	0	***
Voxel Mean Cortical thickness - Glx_A_TissCorrWaterScaled	(Intercept)	Parametric	2.547	0	0.811	0	***
	Glx_A_TissCorrWaterScaled	Parametric	0.011	0.058	0.811	0.189	
	IQ	Parametric	0	0.763	0.811	0.893	
	eTIV	Parametric	0	0.681	0.811	0.852	
	Sex - Female	Parametric	0.012	0.562	0.811	0.852	
	freqShift	Parametric	-0.003	0.808	0.811	0.893	
	s(age)	Smooth	3.122	0	0.811	0	***
Voxel Mean Cortical thickness - Glx_A_CSFWaterScaled	(Intercept)	Parametric	2.519	0	0.813	0	***
	Glx_A_CSFWaterScaled	Parametric	0.015	0.039	0.813	0.132	
	IQ	Parametric	0	0.788	0.813	0.893	
	eTIV	Parametric	0	0.683	0.813	0.852	
	Sex - Female	Parametric	0.011	0.574	0.813	0.852	
	freqShift	Parametric	-0.004	0.765	0.813	0.893	
	s(age)	Smooth	3.112	0	0.813	0	***
Voxel Mean Cortical thickness - tNAA_A_TissCorrWaterScaled	(Intercept)	Parametric	2.59	0	0.807	0	***
	tNAA_A_TissCorrWaterScaled	Parametric	0.011	0.341	0.807	0.852	
	IQ	Parametric	0	0.587	0.807	0.852	
	eTIV	Parametric	0	0.645	0.807	0.852	
	Sex - Female	Parametric	0.009	0.661	0.807	0.852	
	freqShift	Parametric	-0.002	0.874	0.807	0.901	
	s(age)	Smooth	3.424	0	0.807	0	***
Voxel Mean Cortical thickness - tNAA_A_CSFWaterScaled	(Intercept)	Parametric	2.518	0	0.809	0	***
	tNAA_A_CSFWaterScaled	Parametric	0.014	0.216	0.809	0.587	
	IQ	Parametric	0	0.582	0.809	0.852	
	eTIV	Parametric	0	0.657	0.809	0.852	
	Sex - Female	Parametric	0.008	0.704	0.809	0.852	
	freqShift	Parametric	-0.004	0.785	0.809	0.893	
	s(age)	Smooth	3.469	0	0.809	0	***
Voxel Mean Cortical thickness - tCr_A_TissCorrWaterScaled	(Intercept)	Parametric	3.088	0	0.809	0	***
	tCr_A_TissCorrWaterScaled	Parametric	-0.017	0.151	0.809	0.461	
	IQ	Parametric	0	0.682	0.809	0.852	
	eTIV	Parametric	0	0.829	0.809	0.901	
	Sex - Female	Parametric	0.01	0.617	0.809	0.852	
	freqShift	Parametric	0.009	0.513	0.809	0.852	
	s(age)	Smooth	3.266	0	0.809	0	***
Voxel Mean Cortical thickness - tCr_A_CSFWaterScaled	(Intercept)	Parametric	3.069	0	0.808	0	***

	tCr_A_CSFWaterScaled	Parametric	-0.017	0.197	0.808	0.55	
	IQ	Parametric	0	0.665	0.808	0.852	
	eTIV	Parametric	0	0.811	0.808	0.893	
	Sex - Female	Parametric	0.011	0.604	0.808	0.852	
	freqShift	Parametric	0.009	0.533	0.808	0.852	
	s(age)	Smooth	3.263	0	0.808	0	***
Voxel Mean Cortical thickness - tCho_A_TissCorrWaterScaled	(Intercept)	Parametric	2.823	0	0.804	0	***
	tCho_A_TissCorrWaterScaled	Parametric	-0.002	0.961	0.804	0.971	
	IQ	Parametric	0	0.684	0.804	0.852	
	eTIV	Parametric	0	0.634	0.804	0.852	
	Sex - Female	Parametric	0.012	0.549	0.804	0.852	
	freqShift	Parametric	0.003	0.841	0.804	0.901	
	s(age)	Smooth	3.315	0	0.804	0	***
Voxel Mean Cortical thickness - tCho_A_CSFWaterScaled	(Intercept)	Parametric	2.811	0	0.804	0	***
	tCho_A_CSFWaterScaled	Parametric	0.003	0.945	0.804	0.965	
	IQ	Parametric	0	0.692	0.804	0.852	
	eTIV	Parametric	0	0.649	0.804	0.852	
	Sex - Female	Parametric	0.013	0.539	0.804	0.852	
	freqShift	Parametric	0.002	0.866	0.804	0.901	
	s(age)	Smooth	3.319	0	0.804	0	***
Voxel Mean Cortical thickness - mI_A_TissCorrWaterScaled	(Intercept)	Parametric	2.789	0	0.804	0	***
	mI_A_TissCorrWaterScaled	Parametric	0.003	0.803	0.804	0.893	
	IQ	Parametric	0	0.681	0.804	0.852	
	eTIV	Parametric	0	0.628	0.804	0.852	
	Sex - Female	Parametric	0.013	0.528	0.804	0.852	
	freqShift	Parametric	0.002	0.857	0.804	0.901	
	s(age)	Smooth	3.326	0	0.804	0	***
Voxel Mean Cortical thickness - mI_A_CSFWaterScaled	(Intercept)	Parametric	2.771	0	0.804	0	***
	mI_A_CSFWaterScaled	Parametric	0.004	0.694	0.804	0.852	
	IQ	Parametric	0	0.683	0.804	0.852	
	eTIV	Parametric	0	0.625	0.804	0.852	
	Sex - Female	Parametric	0.013	0.519	0.804	0.852	
	freqShift	Parametric	0.002	0.866	0.804	0.901	
	s(age)	Smooth	3.335	0	0.804	0	***
Voxel Mean Cortical thickness - GlxGABAalpharatio (tissue-corrected)	(Intercept)	Parametric	2.868	0	0.806	0	***
	GlxGABAalpharatio	Parametric	-0.016	0.473	0.806	0.852	
	IQ	Parametric	0	0.642	0.806	0.852	
	eTIV	Parametric	0	0.6	0.806	0.852	
	Sex - Female	Parametric	0.012	0.564	0.806	0.852	
	freqShift	Parametric	0	0.980	0.806	0.980	
	s(age)	Smooth	3.382	0	0.806	0	***

Supplementary Table 7. Results from linear regression of PPC voxel mean cortical area predicted by metabolite concentrations, age, IQ, eTIV, frequency shift and sex. Estimate = linear regression predictor beta coefficient. * Indicates significant p values at; '****' $p < 0$ '***' $p < 0.001$ '**' $p < 0.01$ '*' $p < 0.05$. R2 = adjusted R2 obtained from linear modelling.

Metabolite	***Variable***	***Estimate***	***p_value***	***R_squared***	***FDR_p_value***	***Significance_FDR***
Mean Voxel Cortical Area - GABAplus_diff1_AlphaCorrWaterScaled	(Intercept)	0.867	0	0.527	0	***
	GABAplus_diff1_AlphaCorrWaterScaled	0.009	0.128	0.527	0.287	
	age	-0.003	0	0.527	0	***
	Sex	-0.001	0.954	0.527	0.988	
	IQ	0	0.577	0.527	0.788	
	mean_white_surf_area	0	0.012	0.527	0.037	*
	freqShift	0.001	0.906	0.527	0.976	
	Age*sex	0	0.745	0.527	0.86	
Mean Voxel Cortical Area - GABAplus_diff1_TissCorrWaterScaled	(Intercept)	0.872	0	0.522	0	***
	GABAplus_diff1_TissCorrWaterScaled	0.01	0.21	0.522	0.42	
	age	-0.003	0	0.522	0	***
	sex	0	1	0.522	1	
	IQ	0	0.545	0.522	0.78	
	mean_white_surf_area	0	0.013	0.522	0.037	*
	freqShift	0.001	0.816	0.522	0.914	
	Age*sex	0	0.716	0.522	0.845	
Mean Voxel Cortical Area - GABAplus_diff1_CSFWaterScaled	(Intercept)	0.87	0	0.522	0	***
	GABAplus_diff1_CSFWaterScaled	0.011	0.201	0.522	0.409	
	age	-0.003	0	0.522	0	***
	Sex - Female	0	0.979	0.522	0.996	
	IQ	0	0.534	0.522	0.78	

	mean_white_surf_area	0	0.012	0.522	0.037	*
	freqShift	0.001	0.831	0.522	0.922	
	age:Sex - Female	0	0.736	0.522	0.859	
Mean Voxel Cortical Area - Glx_A_TissCorrWaterScaled	(Intercept)	0.85	0	0.526	0	***
	Glx_A_TissCorrWaterScaled	0.003	0.137	0.526	0.302	
	age	-0.002	0	0.526	0.001	**
	Sex - Female	0.001	0.929	0.526	0.982	
	IQ	0	0.422	0.526	0.703	
	mean_white_surf_area	0	0.011	0.526	0.035	*
	freqShift	0.002	0.672	0.526	0.833	
	age:Sex - Female	0	0.662	0.526	0.833	
Mean Voxel Cortical Area - Glx_A_CSFWaterScaled	(Intercept)	0.846	0	0.527	0	***
	Glx_A_CSFWaterScaled	0.003	0.125	0.527	0.285	
	age	-0.002	0	0.527	0.001	**
	Sex - Female	0.001	0.957	0.527	0.988	
	IQ	0	0.409	0.527	0.703	
	mean_white_surf_area	0	0.01	0.527	0.035	*
	freqShift	0.002	0.691	0.527	0.833	
	age:Sex - Female	0	0.686	0.527	0.833	
Mean Voxel Cortical Area - tNAA_A_TissCorrWaterScaled	(Intercept)	0.969	0	0.514	0	***
	tNAA_A_TissCorrWaterScaled	-0.002	0.536	0.514	0.78	
	age	-0.003	0	0.514	0	***
	Sex - Female	0.007	0.654	0.514	0.833	
	IQ	0	0.454	0.514	0.703	
	mean_white_surf_area	0	0.035	0.514	0.091	.
	freqShift	0.005	0.321	0.514	0.599	
	age:Sex - Female	-0.001	0.445	0.514	0.703	
Mean Voxel Cortical Area - tNAA_A_CSFWaterScaled	(Intercept)	0.973	0	0.515	0	***
	tNAA_A_CSFWaterScaled	-0.003	0.501	0.515	0.749	
	age	-0.003	0	0.515	0	***
	Sex - Female	0.008	0.635	0.515	0.829	
	IQ	0	0.459	0.515	0.703	
	mean_white_surf_area	0	0.039	0.515	0.099	.
	freqShift	0.005	0.31	0.515	0.589	
	age:Sex - Female	-0.001	0.433	0.515	0.703	
Mean Voxel Cortical Area - tCr_A_TissCorrWaterScaled	(Intercept)	1.089	0	0.575	0	***
	tCr_A_TissCorrWaterScaled	-0.011	0.001	0.575	0.006	**
	age	-0.002	0	0.575	0	***
	Sex - Female	-0.001	0.962	0.575	0.988	
	IQ	0	0.426	0.575	0.703	
	mean_white_surf_area	0	0.006	0.575	0.021	*
	freqShift	0.008	0.063	0.575	0.154	
	age:Sex - Female	0	0.785	0.575	0.888	
Mean Voxel Cortical Area - tCr_A_CSFWaterScaled	(Intercept)	1.096	0	0.577	0	***
	tCr_A_CSFWaterScaled	-0.013	0.001	0.577	0.005	**
	age	-0.002	0	0.577	0	***
	Sex - Female	0	0.999	0.577	1	
	IQ	0	0.459	0.577	0.703	
	mean_white_surf_area	0	0.006	0.577	0.021	*
	freqShift	0.008	0.055	0.577	0.137	
	age:Sex - Female	0	0.756	0.577	0.864	
Mean Voxel Cortical Area - tCho_A_TissCorrWaterScaled	(Intercept)	0.969	0	0.525	0	***
	tCho_A_TissCorrWaterScaled	-0.019	0.155	0.525	0.329	
	age	-0.002	0	0.525	0	***
	Sex - Female	0.006	0.689	0.525	0.833	
	IQ	0	0.55	0.525	0.78	
	mean_white_surf_area	0	0.017	0.525	0.048	*
	freqShift	0.005	0.243	0.525	0.469	
	age:Sex - Female	-0.001	0.367	0.525	0.643	
Mean Voxel Cortical Area - tCho_A_CSFWaterScaled	(Intercept)	0.97	0	0.526	0	***
	tCho_A_CSFWaterScaled	-0.018	0.146	0.526	0.314	
	age	-0.002	0	0.526	0	***
	Sex - Female	0.006	0.675	0.526	0.833	
	IQ	0	0.564	0.526	0.788	
	mean_white_surf_area	0	0.017	0.526	0.048	*
	freqShift	0.005	0.236	0.526	0.464	
	age:Sex - Female	-0.001	0.356	0.526	0.637	
Mean Voxel Cortical Area - mI_A_TissCorrWaterScaled	(Intercept)	0.984	0	0.531	0	***
	mI_A_TissCorrWaterScaled	-0.006	0.089	0.531	0.208	
	age	-0.003	0	0.531	0	***
	Sex - Female	0.002	0.916	0.531	0.978	
	IQ	0	0.451	0.531	0.703	
	mean_white_surf_area	0	0.017	0.531	0.048	*
	freqShift	0.004	0.358	0.531	0.637	
	age:Sex - Female	0	0.637	0.531	0.829	
Mean Voxel Cortical Area - mI_A_CSFWaterScaled	(Intercept)	0.985	0	0.531	0	***
	mI_A_CSFWaterScaled	-0.006	0.086	0.531	0.204	

	age	-0.003	0	0.531	0	***
	Sex - Female	0.002	0.902	0.531	0.976	
	IQ	0	0.465	0.531	0.703	
	mean_white_surf_area	0	0.018	0.531	0.048	*
	freqShift	0.004	0.354	0.531	0.637	
	age:Sex - Female	0	0.624	0.531	0.829	
Mean Voxel Cortical Area – GlxGABAalpharatio (tissue-corrected)	(Intercept)	0.967	0	0.524	0	***
	GlxGABAalpharatio	-0.01	0.168	0.524	0.348	
	age	-0.003	0	0.524	0	***
	Sex - Female	0.002	0.901	0.524	0.976	
	IQ	0	0.577	0.524	0.788	
	mean_white_surf_area	0	0.012	0.524	0.037	*
	freqShift	0.002	0.707	0.524	0.842	
	age:Sex - Female	0	0.587	0.524	0.792	

Supplementary Table 8. Results from GAM regression of PPC mean cortical area predicted by smooth functions of age, and linear functionals of metabolite concentration, IQ, eTIV and sex as a categorical predictor. Estimate refers to beta coefficients for parametric terms, and the edf value for non-parametric smooth terms (complexity of the smooth function). R2 = estimated R squared values for each GAM. * Indicates significant edf or beta coefficients at; '****' p < 0 '***' p < 0.001 '**' p < 0.01 '*' p < 0.05.

Metabolite	***Variable***	***TermType***	***p_value***	***Adjusted_R2***	***FDR_p_value***	***Significance_FDR***	***estimate***
Mean Voxel Cortical Area - GABAplus_diff1_AlphaCorrWaterScaled	(Intercept)	Parametric	0	0.582	0	***	0.824
	GABAplus_diff1_AlphaCorrWaterScaled	Parametric	0.658	0.582	0.877		0.002
	IQ	Parametric	0.297	0.582	0.485		0
	mean_white_surf_area	Parametric	0.063	0.582	0.168		0
	Sex - Female	Parametric	0.971	0.582	0.994		0
	freqShift	Parametric	0.747	0.582	0.927		0.001
	s(age)	Smooth	0	0.582	0	***	3.997
Mean Voxel Cortical Area - GABAplus_diff1_TissCorrWaterScaled	(Intercept)	Parametric	0	0.584	0	***	0.833
	GABAplus_diff1_TissCorrWaterScaled	Parametric	0.878	0.584	0.994		0.001
	IQ	Parametric	0.287	0.584	0.485		0
	mean_white_surf_area	Parametric	0.069	0.584	0.168		0
	Sex - Female	Parametric	0.928	0.584	0.994		0.001
	freqShift	Parametric	0.662	0.584	0.877		0.002
	s(age)	Smooth	0	0.584	0	***	4.131
Mean Voxel Cortical Area - GABAplus_diff1_CSFWaterScaled	(Intercept)	Parametric	0	0.583	0	***	0.831
	GABAplus_diff1_CSFWaterScaled	Parametric	0.827	0.583	0.994		0.002
	IQ	Parametric	0.287	0.583	0.485		0
	mean_white_surf_area	Parametric	0.068	0.583	0.168		0
	Sex - Female	Parametric	0.937	0.583	0.994		0
	freqShift	Parametric	0.682	0.583	0.88		0.002
	s(age)	Smooth	0	0.583	0	***	4.107
Mean Voxel Cortical Area - Glx_A_TissCorrWaterScaled	(Intercept)	Parametric	0	0.587	0	***	0.813
	Glx_A_TissCorrWaterScaled	Parametric	0.513	0.587	0.729		0.001
	IQ	Parametric	0.263	0.587	0.485		0
	mean_white_surf_area	Parametric	0.061	0.587	0.168		0
	Sex - Female	Parametric	0.934	0.587	0.994		0
	freqShift	Parametric	0.707	0.587	0.9		0.002
	s(age)	Smooth	0	0.587	0	***	4.219
Mean Voxel Cortical Area - Glx_A_CSFWaterScaled	(Intercept)	Parametric	0	0.588	0	***	0.81
	Glx_A_CSFWaterScaled	Parametric	0.464	0.588	0.668		0.002
	IQ	Parametric	0.258	0.588	0.485		0
	mean_white_surf_area	Parametric	0.059	0.588	0.168		0
	Sex - Female	Parametric	0.941	0.588	0.994		0
	freqShift	Parametric	0.725	0.588	0.911		0.001
	s(age)	Smooth	0	0.588	0	***	4.234
Mean Voxel Cortical Area - tNAA_A_TissCorrWaterScaled	(Intercept)	Parametric	0	0.584	0	***	0.829
	tNAA_A_TissCorrWaterScaled	Parametric	0.879	0.584	0.994		0.001
	IQ	Parametric	0.296	0.584	0.485		0
	mean_white_surf_area	Parametric	0.076	0.584	0.17		0
	Sex - Female	Parametric	0.95	0.584	0.994		0
	freqShift	Parametric	0.659	0.584	0.877		0.002
	s(age)	Smooth	0	0.584	0	***	4.201
Mean Voxel Cortical Area - tNAA_A_CSFWaterScaled	(Intercept)	Parametric	0	0.585	0	***	0.825
	tNAA_A_CSFWaterScaled	Parametric	0.828	0.585	0.994		0.001
	IQ	Parametric	0.295	0.585	0.485		0
	mean_white_surf_area	Parametric	0.074	0.585	0.169		0
	Sex - Female	Parametric	0.966	0.585	0.994		0
	freqShift	Parametric	0.68	0.585	0.88		0.002
	s(age)	Smooth	0	0.585	0	***	4.21
Mean Voxel Cortical Area - tCr_A_TissCorrWaterScaled	(Intercept)	Parametric	0	0.622	0	***	0.98

	tCr_A_TissCorrWaterScaled	Parametric	0.006	0.622	0.019	*	-0.009
	IQ	Parametric	0.25	0.622	0.485		0
	mean_white_surf_area	Parametric	0.029	0.622	0.09	.	0
	Sex - Female	Parametric	0.996	0.622	0.996		0
	freqShift	Parametric	0.144	0.622	0.307		0.006
	s(age)	Smooth	0	0.622	0	***	4.052
Mean Voxel Cortical Area - tCr_A_CSFWaterScaled	(Intercept)	Parametric	0	0.622	0	***	0.985
	tCr_A_CSFWaterScaled	Parametric	0.006	0.622	0.019	*	-0.01
	IQ	Parametric	0.271	0.622	0.485		0
	mean_white_surf_area	Parametric	0.029	0.622	0.09	.	0
	Sex - Female	Parametric	0.982	0.622	0.994		0
	freqShift	Parametric	0.133	0.622	0.29		0.006
	s(age)	Smooth	0	0.622	0	***	3.989
Mean Voxel Cortical Area - tCho_A_TissCorrWaterScaled	(Intercept)	Parametric	0	0.592	0	***	0.876
	tCho_A_TissCorrWaterScaled	Parametric	0.262	0.592	0.485		-0.014
	IQ	Parametric	0.325	0.592	0.513		0
	mean_white_surf_area	Parametric	0.071	0.592	0.168		0
	Sex - Female	Parametric	0.909	0.592	0.994		-0.001
	freqShift	Parametric	0.42	0.592	0.614		0.003
	s(age)	Smooth	0	0.592	0	***	4.248
Mean Voxel Cortical Area - tCho_A_CSFWaterScaled	(Intercept)	Parametric	0	0.592	0	***	0.876
	tCho_A_CSFWaterScaled	Parametric	0.267	0.592	0.485		-0.012
	IQ	Parametric	0.333	0.592	0.517		0
	mean_white_surf_area	Parametric	0.072	0.592	0.168		0
	Sex - Female	Parametric	0.911	0.592	0.994		-0.001
	freqShift	Parametric	0.418	0.592	0.614		0.003
	s(age)	Smooth	0	0.592	0	***	4.232
Mean Voxel Cortical Area - mI_A_TissCorrWaterScaled	(Intercept)	Parametric	0	0.586	0	***	0.87
	mI_A_TissCorrWaterScaled	Parametric	0.349	0.586	0.534		-0.003
	IQ	Parametric	0.269	0.586	0.485		0
	mean_white_surf_area	Parametric	0.066	0.586	0.168		0
	Sex - Female	Parametric	0.984	0.586	0.994		0
	freqShift	Parametric	0.544	0.586	0.751		0.002
	s(age)	Smooth	0	0.586	0	***	4.028
Mean Voxel Cortical Area - mI_A_CSFWaterScaled	(Intercept)	Parametric	0	0.586	0	***	0.87
	mI_A_CSFWaterScaled	Parametric	0.361	0.586	0.544		-0.003
	IQ	Parametric	0.275	0.586	0.485		0
	mean_white_surf_area	Parametric	0.067	0.586	0.168		0
	Sex - Female	Parametric	0.981	0.586	0.994		0
	freqShift	Parametric	0.542	0.586	0.751		0.002
	s(age)	Smooth	0	0.586	0	***	4.015
Mean Voxel Cortical Area – GlxGABAalpharatio (tissue-corrected)	(Intercept)	Parametric	0	0.588	0	***	0.869
	GlxGABAalpharatio	Parametric	0.221	0.588	0.461		-0.008
	IQ	Parametric	0.32	0.588	0.513		0
	mean_white_surf_area	Parametric	0.055	0.588	0.163		0
	Sex - Female	Parametric	0.959	0.588	0.994		0
	freqShift	Parametric	0.875	0.588	0.994		0.001
	s(age)	Smooth	0	0.588	0	***	3.913

Supplementary Table 9. Results from linear regression of PPC voxel mean cortical volume predicted by metabolite concentrations, age, IQ, eTIV, frequency shift and sex. Estimate = linear regression predictor beta coefficient. * Indicates significant p values at; '****' $p < 0$ '***' $p < 0.001$ '**' $p < 0.01$ '*' $p < 0.05$. R2 = adjusted R2 obtained from linear modelling.

Metabolite	*Variable*	*Estimate*	*p_value*	*R_squared*	*FDR_p_value*	*Significance_FDR*
Mean Voxel Cortical Volume - GABAplus_diff1_AlphaCorrWaterScaled	(Intercept)	2.315	0	0.769	0	***
	GABAplus_diff1_AlphaCorrWaterScaled	0.041	0.055	0.769	0.159	
	age	-0.022	0	0.769	0	***
	IQ	-0.001	0.424	0.769	0.812	
	eTIV	0	0.86	0.769	0.98	
	Sex - Female	-0.006	0.806	0.769	0.98	
	freqShift	0.001	0.943	0.769	0.98	
Mean Voxel Cortical Volume - GABAplus_diff1_TissCorrWaterScaled	(Intercept)	2.307	0	0.767	0	***
	GABAplus_diff1_TissCorrWaterScaled	0.052	0.071	0.767	0.189	
	age	-0.022	0	0.767	0	***
	IQ	-0.001	0.456	0.767	0.812	
	eTIV	0	0.907	0.767	0.98	
	Sex - Female	-0.005	0.83	0.767	0.98	
	freqShift	0.002	0.902	0.767	0.98	
Mean Voxel Cortical Volume - GABAplus_diff1_CSFWaterScaled	(Intercept)	2.291	0	0.768	0	***
	GABAplus_diff1_CSFWaterScaled	0.059	0.06	0.768	0.164	
	age	-0.022	0	0.768	0	***
	IQ	-0.001	0.47	0.768	0.822	
	eTIV	0	0.911	0.768	0.98	
	Sex - Female	-0.006	0.818	0.768	0.98	
	freqShift	0.002	0.933	0.768	0.98	

Mean Voxel Cortical Volume - Glx_A_TissCorrWaterScaled	(Intercept)	2.071	0	0.78	0	***
	Glx_A_TissCorrWaterScaled	0.019	0.007	0.78	0.022	*
	age	-0.018	0	0.78	0	***
	IQ	0	0.666	0.78	0.98	
	eTIV	0	0.966	0.78	0.98	
	Sex - Female	-0.003	0.917	0.78	0.98	
	freqShift	0.004	0.814	0.78	0.98	
Mean Voxel Cortical Volume - Glx_A_CSFWaterScaled	(Intercept)	2.037	0	0.782	0	***
	Glx_A_CSFWaterScaled	0.024	0.005	0.782	0.015	*
	age	-0.018	0	0.782	0	***
	IQ	0	0.702	0.782	0.98	
	eTIV	0	0.97	0.782	0.98	
	Sex - Female	-0.003	0.903	0.782	0.98	
	freqShift	0.003	0.855	0.782	0.98	
Mean Voxel Cortical Volume - tNAA_A_TissCorrWaterScaled	(Intercept)	2.939	0	0.761	0	***
	tNAA_A_TissCorrWaterScaled	-0.016	0.269	0.761	0.599	
	age	-0.022	0	0.761	0	***
	IQ	0	0.619	0.761	0.956	
	eTIV	0	0.893	0.761	0.98	
	Sex - Female	0.001	0.961	0.761	0.98	
	freqShift	0.022	0.225	0.761	0.55	
Mean Voxel Cortical Volume - tNAA_A_CSFWaterScaled	(Intercept)	2.92	0	0.76	0	***
	tNAA_A_CSFWaterScaled	-0.014	0.303	0.76	0.659	
	age	-0.022	0	0.76	0	***
	IQ	-0.001	0.585	0.76	0.924	
	eTIV	0	0.883	0.76	0.98	
	Sex - Female	0.001	0.966	0.76	0.98	
	freqShift	0.022	0.233	0.76	0.557	
Mean Voxel Cortical Volume - tCr_A_TissCorrWaterScaled	(Intercept)	3.288	0	0.788	0	***
	tCr_A_TissCorrWaterScaled	-0.045	0.001	0.788	0.005	**
	age	-0.02	0	0.788	0	***
	IQ	-0.001	0.539	0.788	0.88	
	eTIV	0	0.628	0.788	0.956	
	Sex - Female	-0.007	0.754	0.788	0.98	
	freqShift	0.033	0.058	0.788	0.161	
Mean Voxel Cortical Volume - tCr_A_CSFWaterScaled	(Intercept)	3.303	0	0.788	0	***
	tCr_A_CSFWaterScaled	-0.05	0.002	0.788	0.005	**
	age	-0.02	0	0.788	0	***
	IQ	-0.001	0.498	0.788	0.857	
	eTIV	0	0.634	0.788	0.956	
	Sex - Female	-0.007	0.774	0.788	0.98	
	freqShift	0.033	0.054	0.788	0.159	
Mean Voxel Cortical Volume - tCho_A_TissCorrWaterScaled	(Intercept)	2.73	0	0.76	0	***
	tCho_A_TissCorrWaterScaled	-0.053	0.321	0.76	0.663	
	age	-0.021	0	0.76	0	***
	IQ	-0.001	0.446	0.76	0.812	
	eTIV	0	0.798	0.76	0.98	
	Sex - Female	-0.008	0.766	0.76	0.98	
	freqShift	0.02	0.257	0.76	0.589	
Mean Voxel Cortical Volume - tCho_A_CSFWaterScaled	(Intercept)	2.729	0	0.76	0	***
	tCho_A_CSFWaterScaled	-0.047	0.334	0.76	0.669	
	age	-0.021	0	0.76	0	***
	IQ	-0.001	0.439	0.76	0.812	
	eTIV	0	0.795	0.76	0.98	
	Sex - Female	-0.007	0.771	0.76	0.98	
	freqShift	0.02	0.259	0.76	0.589	
Mean Voxel Cortical Volume - mI_A_TissCorrWaterScaled	(Intercept)	2.859	0	0.767	0	***
	mI_A_TissCorrWaterScaled	-0.025	0.083	0.767	0.213	
	age	-0.023	0	0.767	0	***
	IQ	-0.001	0.555	0.767	0.892	
	eTIV	0	0.98	0.767	0.98	
	Sex - Female	-0.006	0.797	0.767	0.98	
	freqShift	0.017	0.325	0.767	0.663	
Mean Voxel Cortical Volume - mI_A_CSFWaterScaled	(Intercept)	2.857	0	0.766	0	***
	mI_A_CSFWaterScaled	-0.023	0.091	0.766	0.229	
	age	-0.023	0	0.766	0	***
	IQ	-0.001	0.536	0.766	0.88	
	eTIV	0	0.97	0.766	0.98	
	Sex - Female	-0.006	0.805	0.766	0.98	
	freqShift	0.017	0.323	0.766	0.663	
Mean Voxel Cortical Volume - GlxGABAalpharatio (tissue-corrected)	(Intercept)	2.705	0	0.759	0	***
	GlxGABAalpharatio	-0.022	0.42	0.759	0.812	
	age	-0.023	0	0.759	0	***
	IQ	-0.001	0.449	0.759	0.812	
	eTIV	0	0.906	0.759	0.98	
	Sex - Female	-0.006	0.813	0.759	0.98	
	freqShift	0.011	0.53	0.759	0.88	

Supplementary Table 10. Results from GAM regression of PPC mean cortical volume predicted by smooth functions of age, and linear functionals of metabolite concentration, IQ, eTIV and sex as a categorical predictor. Estimate refers to beta coefficients for parametric terms, and the edf value for non-parametric smooth terms (complexity of the smooth function). R2 = estimated R squared values for each GAM. * Indicates significant edf or beta coefficients at; '****' $p < 0$ '***' $p < 0.001$ '**' $p < 0.01$ '*' $p < 0.05$.

Metabolite	*Variable*	*TermType*	*p_value*	*Adjusted_R2*	*FDR_p_value*	*Significance_FDR*	*estimate*
Mean Voxel Cortical Volume - GABAplus_diff1_AlphaCorrWaterScaled	(Intercept)	Parametric	0	0.846	0	***	2.031
	GABAplus_diff1_AlphaCorrWaterScaled	Parametric	0.502	0.846	0.928		0.012
	IQ	Parametric	0.953	0.846	0.993		0
	eTIV	Parametric	0.65	0.846	0.947		0
	Sex - Female	Parametric	0.291	0.846	0.648		0.021
	freqShift	Parametric	0.768	0.846	0.947		0.004
	s(age)	Smooth	0	0.846	0	***	4.141
Mean Voxel Cortical Volume - GABAplus_diff1_TissCorrWaterScaled	(Intercept)	Parametric	0	0.846	0	***	2.046
	GABAplus_diff1_TissCorrWaterScaled	Parametric	0.605	0.846	0.947		0.012
	IQ	Parametric	0.935	0.846	0.993		0
	eTIV	Parametric	0.661	0.846	0.947		0
	Sex - Female	Parametric	0.281	0.846	0.648		0.022
	freqShift	Parametric	0.721	0.846	0.947		0.005
	s(age)	Smooth	0	0.846	0	***	4.155
Mean Voxel Cortical Volume - GABAplus_diff1_CSFWaterScaled	(Intercept)	Parametric	0	0.846	0	***	2.028
	GABAplus_diff1_CSFWaterScaled	Parametric	0.522	0.846	0.938		0.017
	IQ	Parametric	0.936	0.846	0.993		0
	eTIV	Parametric	0.66	0.846	0.947		0
	Sex - Female	Parametric	0.287	0.846	0.648		0.021
	freqShift	Parametric	0.759	0.846	0.947		0.005
	s(age)	Smooth	0	0.846	0	***	4.157
Mean Voxel Cortical Volume - Glx_A_TissCorrWaterScaled	(Intercept)	Parametric	0	0.848	0	***	1.929
	Glx_A_TissCorrWaterScaled	Parametric	0.179	0.848	0.515		0.008
	IQ	Parametric	0.836	0.848	0.98		0
	eTIV	Parametric	0.706	0.848	0.947		0
	Sex - Female	Parametric	0.271	0.848	0.648		0.022
	freqShift	Parametric	0.773	0.848	0.947		0.004
	s(age)	Smooth	0	0.848	0	***	4.107
Mean Voxel Cortical Volume - Glx_A_CSFWaterScaled	(Intercept)	Parametric	0	0.849	0	***	1.903
	Glx_A_CSFWaterScaled	Parametric	0.132	0.849	0.419		0.01
	IQ	Parametric	0.816	0.849	0.975		0
	eTIV	Parametric	0.708	0.849	0.947		0
	Sex - Female	Parametric	0.276	0.849	0.648		0.022
	freqShift	Parametric	0.809	0.849	0.975		0.003
	s(age)	Smooth	0	0.849	0	***	4.11
Mean Voxel Cortical Volume - tNAA_A_TissCorrWaterScaled	(Intercept)	Parametric	0	0.846	0	***	1.998
	tNAA_A_TissCorrWaterScaled	Parametric	0.608	0.846	0.947		0.006
	IQ	Parametric	0.974	0.846	0.993		0
	eTIV	Parametric	0.672	0.846	0.947		0
	Sex - Female	Parametric	0.313	0.846	0.682		0.021
	freqShift	Parametric	0.7	0.846	0.947		0.006
	s(age)	Smooth	0	0.846	0	***	4.237
Mean Voxel Cortical Volume - tNAA_A_CSFWaterScaled	(Intercept)	Parametric	0	0.847	0	***	1.946
	tNAA_A_CSFWaterScaled	Parametric	0.468	0.847	0.918		0.008
	IQ	Parametric	0.984	0.847	0.993		0
	eTIV	Parametric	0.678	0.847	0.947		0
	Sex - Female	Parametric	0.334	0.847	0.696		0.02
	freqShift	Parametric	0.76	0.847	0.947		0.004
	s(age)	Smooth	0	0.847	0	***	4.267
Mean Voxel Cortical Volume - tCr_A_TissCorrWaterScaled	(Intercept)	Parametric	0	0.858	0	***	2.571
	tCr_A_TissCorrWaterScaled	Parametric	0.012	0.858	0.042	*	-0.028
	IQ	Parametric	0.896	0.858	0.993		0
	eTIV	Parametric	0.987	0.858	0.993		0
	Sex - Female	Parametric	0.342	0.858	0.697		0.018
	freqShift	Parametric	0.158	0.858	0.474		0.02
	s(age)	Smooth	0	0.858	0	***	4.138
Mean Voxel Cortical Volume - tCr_A_CSFWaterScaled	(Intercept)	Parametric	0	0.856	0	***	2.57
	tCr_A_CSFWaterScaled	Parametric	0.016	0.856	0.053	.	-0.031
	IQ	Parametric	0.931	0.856	0.993		0
	eTIV	Parametric	0.993	0.856	0.993		0
	Sex - Female	Parametric	0.333	0.856	0.696		0.019
	freqShift	Parametric	0.16	0.856	0.474		0.02
	s(age)	Smooth	0	0.856	0	***	4.115
Mean Voxel Cortical Volume - tCho_A_TissCorrWaterScaled	(Intercept)	Parametric	0	0.846	0	***	2.159
	tCho_A_TissCorrWaterScaled	Parametric	0.679	0.846	0.947		-0.017
	IQ	Parametric	0.935	0.846	0.993		0
	eTIV	Parametric	0.619	0.846	0.947		0
	Sex - Female	Parametric	0.29	0.846	0.648		0.021

	freqShift	Parametric	0.487	0.846	0.928		0.01
	s(age)	Smooth	0	0.846	0	***	4.187
Mean Voxel Cortical Volume - tCho_A_CSFWaterScaled	(Intercept)	Parametric	0	0.845	0	***	2.152
	tCho_A_CSFWaterScaled	Parametric	0.74	0.845	0.947		-0.013
	IQ	Parametric	0.933	0.845	0.993		0
	eTIV	Parametric	0.629	0.845	0.947		0
	Sex - Female	Parametric	0.286	0.845	0.648		0.022
	freqShift	Parametric	0.5	0.845	0.928		0.01
	s(age)	Smooth	0	0.845	0	***	4.186
Mean Voxel Cortical Volume - mI_A_TissCorrWaterScaled	(Intercept)	Parametric	0	0.845	0	***	2.154
	mI_A_TissCorrWaterScaled	Parametric	0.754	0.845	0.947		-0.004
	IQ	Parametric	0.899	0.845	0.993		0
	eTIV	Parametric	0.675	0.845	0.947		0
	Sex - Female	Parametric	0.28	0.845	0.648		0.022
	freqShift	Parametric	0.532	0.845	0.938		0.008
	s(age)	Smooth	0	0.845	0	***	4.169
Mean Voxel Cortical Volume - mI_A_CSFWaterScaled	(Intercept)	Parametric	0	0.845	0	***	2.142
	mI_A_CSFWaterScaled	Parametric	0.84	0.845	0.98		-0.002
	IQ	Parametric	0.904	0.845	0.993		0
	eTIV	Parametric	0.672	0.845	0.947		0
	Sex - Female	Parametric	0.275	0.845	0.648		0.022
	freqShift	Parametric	0.536	0.845	0.938		0.008
	s(age)	Smooth	0	0.845	0	***	4.174
Mean Voxel Cortical Volume - GlxGABAalpharatio (tissue-crorrected)	(Intercept)	Parametric	0	0.848	0	***	2.181
	GlxGABAalpharatio	Parametric	0.381	0.848	0.761		-0.019
	IQ	Parametric	0.981	0.848	0.993		0
	eTIV	Parametric	0.613	0.848	0.947		0
	Sex - Female	Parametric	0.283	0.848	0.648		0.021
	freqShift	Parametric	0.738	0.848	0.947		0.005
	s(age)	Smooth	0	0.848	0	***	4.266

Supplementary Table 11. Results from linear regression of PPC voxel mean cortical LGI predicted by metabolite concentrations, age, IQ, eTIV, frequency shift and sex. Estimate = linear regression predictor beta coefficient. * Indicates significant p values at; '****' p < 0 '***' p < 0.001 '**' p < 0.01 '*' p < 0.05. R2 = adjusted R2 obtained from linear modelling.

Metabolite	***Variable***	***Estimate***	***p_value***	***R_squared***	***FDR_p_value***	***Significance_FDR***
Mean Voxel Cortical LGI - GABAplus_diff1_AlphaCorrWaterScaled	(Intercept)	2.526	0	0.346	0	***
	GABAplus_diff1_AlphaCorrWaterScaled	0.026	0.432	0.346	0.794	
	age	-0.009	0.001	0.346	0.005	**
	Sex - Female	-0.006	0.943	0.346	0.99	
	IQ	0	0.932	0.346	0.99	
	eTIV	0	0	0.346	0.001	***
	freqShift	0.041	0.135	0.346	0.28	
	age:Sex - Female	0	0.965	0.346	0.99	
Mean Voxel Cortical LGI - GABAplus_diff1_TissCorrWaterScaled	(Intercept)	2.506	0	0.346	0	***
	GABAplus_diff1_TissCorrWaterScaled	0.035	0.426	0.346	0.794	
	age	-0.009	0.001	0.346	0.005	**
	Sex - Female	-0.007	0.936	0.346	0.99	
	IQ	0	0.917	0.346	0.99	
	eTIV	0	0	0.346	0.001	***
	freqShift	0.041	0.135	0.346	0.28	
	age:Sex - Female	0	0.953	0.346	0.99	
Mean Voxel Cortical LGI - GABAplus_diff1_CSFWaterScaled	(Intercept)	2.51	0	0.346	0	***
	GABAplus_diff1_CSFWaterScaled	0.037	0.44	0.346	0.794	
	age	-0.009	0.001	0.346	0.005	**
	Sex - Female	-0.008	0.932	0.346	0.99	
	IQ	0	0.909	0.346	0.99	
	eTIV	0	0	0.346	0.001	***
	freqShift	0.041	0.135	0.346	0.28	
	age:Sex - Female	0	0.95	0.346	0.99	
Mean Voxel Cortical LGI - Glx_A_TissCorrWaterScaled	(Intercept)	1.905	0	0.402	0	***
	Glx_A_TissCorrWaterScaled	0.028	0.008	0.402	0.022	*
	age	-0.002	0.472	0.402	0.814	
	Sex - Female	-0.023	0.787	0.402	0.99	
	IQ	0.001	0.671	0.402	0.99	
	eTIV	0	0	0.402	0.001	***
	freqShift	0.034	0.177	0.402	0.36	
	age:Sex - Female	0.002	0.739	0.402	0.99	
Mean Voxel Cortical LGI - Glx_A_CSFWaterScaled	(Intercept)	1.893	0	0.402	0	***
	Glx_A_CSFWaterScaled	0.033	0.008	0.402	0.022	*
	age	-0.002	0.472	0.402	0.814	
	Sex - Female	-0.026	0.754	0.402	0.99	
	IQ	0.001	0.649	0.402	0.99	
	eTIV	0	0	0.402	0.001	***
	freqShift	0.033	0.185	0.402	0.369	
	age:Sex - Female	0.002	0.711	0.402	0.99	

Mean Voxel Cortical LGI - tNAA_A_TissCorrWaterScaled	(Intercept)	2.394	0	0.346	0	***
	tNAA_A_TissCorrWaterScaled	0.017	0.427	0.346	0.794	
	age	-0.008	0.002	0.346	0.005	**
	Sex - Female	0.004	0.963	0.346	0.99	
	IQ	0	0.99	0.346	0.99	
	eTIV	0	0	0.346	0.001	***
	freqShift	0.043	0.105	0.346	0.232	
	age:Sex - Female	-0.001	0.866	0.346	0.99	
Mean Voxel Cortical LGI - tNAA_A_CSFWaterScaled	(Intercept)	2.42	0	0.345	0	***
	tNAA_A_CSFWaterScaled	0.015	0.469	0.345	0.814	
	age	-0.008	0.002	0.345	0.005	**
	Sex - Female	0.003	0.972	0.345	0.99	
	IQ	0	0.982	0.345	0.99	
	eTIV	0	0	0.345	0.001	***
	freqShift	0.044	0.102	0.345	0.23	
	age:Sex - Female	-0.001	0.879	0.345	0.99	
Mean Voxel Cortical LGI - tCr_A_TissCorrWaterScaled	(Intercept)	2.665	0	0.341	0	***
	tCr_A_TissCorrWaterScaled	0.004	0.86	0.341	0.99	
	age	-0.009	0.003	0.341	0.009	**
	Sex - Female	0.013	0.882	0.341	0.99	
	IQ	0	0.898	0.341	0.99	
	eTIV	0	0	0.341	0.001	***
	freqShift	0.049	0.068	0.341	0.166	
	age:Sex - Female	-0.001	0.847	0.341	0.99	
Mean Voxel Cortical LGI - tCr_A_CSFWaterScaled	(Intercept)	2.674	0	0.341	0	***
	tCr_A_CSFWaterScaled	0.003	0.887	0.341	0.99	
	age	-0.008	0.003	0.341	0.009	**
	Sex - Female	0.012	0.887	0.341	0.99	
	IQ	0	0.895	0.341	0.99	
	eTIV	0	0	0.341	0.001	***
	freqShift	0.049	0.068	0.341	0.166	
	age:Sex - Female	-0.001	0.851	0.341	0.99	
Mean Voxel Cortical LGI - tCho_A_TissCorrWaterScaled	(Intercept)	2.637	0	0.343	0	***
	tCho_A_TissCorrWaterScaled	0.042	0.587	0.343	0.99	
	age	-0.009	0.004	0.343	0.011	*
	Sex - Female	0.009	0.914	0.343	0.99	
	IQ	0	0.876	0.343	0.99	
	eTIV	0	0	0.343	0.001	**
	freqShift	0.046	0.078	0.343	0.18	
	age:Sex - Female	-0.001	0.898	0.343	0.99	
Mean Voxel Cortical LGI - tCho_A_CSFWaterScaled	(Intercept)	2.639	0	0.343	0	***
	tCho_A_CSFWaterScaled	0.037	0.599	0.343	0.99	
	age	-0.009	0.004	0.343	0.011	*
	Sex - Female	0.009	0.918	0.343	0.99	
	IQ	0	0.871	0.343	0.99	
	eTIV	0	0	0.343	0.001	**
	freqShift	0.046	0.079	0.343	0.18	
	age:Sex - Female	-0.001	0.901	0.343	0.99	
Mean Voxel Cortical LGI - mI_A_TissCorrWaterScaled	(Intercept)	2.528	0	0.349	0	***
	mI_A_TissCorrWaterScaled	0.02	0.335	0.349	0.659	
	age	-0.008	0.003	0.349	0.01	*
	Sex - Female	0.024	0.785	0.349	0.99	
	IQ	0	0.94	0.349	0.99	
	eTIV	0	0	0.349	0.001	***
	freqShift	0.049	0.053	0.349	0.135	
	age:Sex - Female	-0.002	0.747	0.349	0.99	
Mean Voxel Cortical LGI - mI_A_CSFWaterScaled	(Intercept)	2.529	0	0.349	0	***
	mI_A_CSFWaterScaled	0.019	0.346	0.349	0.668	
	age	-0.008	0.003	0.349	0.01	*
	Sex - Female	0.023	0.795	0.349	0.99	
	IQ	0	0.928	0.349	0.99	
	eTIV	0	0	0.349	0.001	***
	freqShift	0.049	0.053	0.349	0.135	
	age:Sex - Female	-0.002	0.756	0.349	0.99	
Mean Voxel Cortical LGI - GlxGABAalpharatio (tissue-corrected)	(Intercept)	2.775	0	0.342	0	***
	GlxGABAalpharatio	-0.015	0.706	0.342	0.99	
	age	-0.009	0.002	0.342	0.008	**
	Sex - Female	0.006	0.943	0.342	0.99	
	IQ	0	0.915	0.342	0.99	
	eTIV	0	0	0.342	0.001	***
	freqShift	0.047	0.075	0.342	0.179	
	age:Sex - Female	-0.001	0.907	0.342	0.99	

Supplementary Table 12. Results from GAM regression of PPC mean cortical LGI predicted by smooth functions of age, and linear functionals of metabolite concentration, IQ, eTIV and sex as a categorical predictor. Estimate refers to beta coefficients for parametric terms, and the edf value for non-parametric smooth terms (complexity of the smooth function). R2 = estimated R squared values for each GAM. * Indicates significant edf or beta coefficients at; '****' p < 0 '***' p < 0.001 '**' p < 0.01 '*' p < 0.05.

Metabolite	Variable	TermType	p_value	Adjusted_R2	FDR_p_value	Significance_FDR	estimate
Mean Voxel Cortical LGI - GABAplus_diff1_AlphaCorrWaterScaled	(Intercept)	Parametric	0	0.292	0	***	2.386
	GABAplus_diff1_AlphaCorrWaterScaled	Parametric	0.419	0.292	0.673		0.026
	IQ	Parametric	0.927	0.292	0.995		0
	eTIV	Parametric	0	0.292	0.001	***	0
	Sex - Female	Parametric	0.939	0.292	0.995		-0.003
	freqShift	Parametric	0.129	0.292	0.234		0.041
	s(age)	Smooth	0	0.292	0.001	***	1
Mean Voxel Cortical LGI - GABAplus_diff1_TissCorrWaterScaled	(Intercept)	Parametric	0	0.292	0	***	2.37
	GABAplus_diff1_TissCorrWaterScaled	Parametric	0.414	0.292	0.673		0.034
	IQ	Parametric	0.911	0.292	0.995		0
	eTIV	Parametric	0	0.292	0.001	***	0
	Sex - Female	Parametric	0.949	0.292	0.995		-0.002
	freqShift	Parametric	0.128	0.292	0.234		0.041
	s(age)	Smooth	0	0.292	0.001	***	1
Mean Voxel Cortical LGI - GABAplus_diff1_CSFWaterScaled	(Intercept)	Parametric	0	0.292	0	***	2.374
	GABAplus_diff1_CSFWaterScaled	Parametric	0.427	0.292	0.675		0.036
	IQ	Parametric	0.902	0.292	0.995		0
	eTIV	Parametric	0	0.292	0.001	***	0
	Sex - Female	Parametric	0.944	0.292	0.995		-0.003
	freqShift	Parametric	0.128	0.292	0.234		0.042
	s(age)	Smooth	0	0.292	0.001	***	1
Mean Voxel Cortical LGI - Glx_A_TissCorrWaterScaled	(Intercept)	Parametric	0	0.352	0	***	1.886
	Glx_A_TissCorrWaterScaled	Parametric	0.008	0.352	0.019	*	0.028
	IQ	Parametric	0.646	0.352	0.918		0.001
	eTIV	Parametric	0	0.352	0.001	***	0
	Sex - Female	Parametric	0.94	0.352	0.995		0.003
	freqShift	Parametric	0.169	0.352	0.301		0.034
	s(age)	Smooth	0.507	0.352	0.772		1
Mean Voxel Cortical LGI - Glx_A_CSFWaterScaled	(Intercept)	Parametric	0	0.351	0	***	1.878
	Glx_A_CSFWaterScaled	Parametric	0.008	0.351	0.02	*	0.032
	IQ	Parametric	0.623	0.351	0.897		0.001
	eTIV	Parametric	0	0.351	0.001	***	0
	Sex - Female	Parametric	0.956	0.351	0.995		0.002
	freqShift	Parametric	0.175	0.351	0.307		0.034
	s(age)	Smooth	0.512	0.351	0.772		1
Mean Voxel Cortical LGI - tNAA_A_TissCorrWaterScaled	(Intercept)	Parametric	0	0.293	0	***	2.247
	tNAA_A_TissCorrWaterScaled	Parametric	0.419	0.293	0.673		0.017
	IQ	Parametric	0.979	0.293	0.995		0
	eTIV	Parametric	0	0.293	0.001	***	0
	Sex - Female	Parametric	0.808	0.293	0.995		-0.009
	freqShift	Parametric	0.104	0.293	0.201		0.043
	s(age)	Smooth	0	0.293	0.001	***	1.041
Mean Voxel Cortical LGI - tNAA_A_CSFWaterScaled	(Intercept)	Parametric	0	0.291	0	***	2.275
	tNAA_A_CSFWaterScaled	Parametric	0.462	0.291	0.718		0.015
	IQ	Parametric	0.995	0.291	0.995		0
	eTIV	Parametric	0	0.291	0.001	***	0
	Sex - Female	Parametric	0.812	0.291	0.995		-0.009
	freqShift	Parametric	0.101	0.291	0.198		0.044
	s(age)	Smooth	0	0.291	0.001	***	1.014
Mean Voxel Cortical LGI - tCr_A_TissCorrWaterScaled	(Intercept)	Parametric	0	0.286	0	***	2.53
	tCr_A_TissCorrWaterScaled	Parametric	0.877	0.286	0.995		0.003
	IQ	Parametric	0.914	0.286	0.995		0
	eTIV	Parametric	0	0.286	0.001	***	0
	Sex - Female	Parametric	0.948	0.286	0.995		-0.002
	freqShift	Parametric	0.065	0.286	0.139		0.049
	s(age)	Smooth	0.001	0.286	0.002	**	1
Mean Voxel Cortical LGI - tCr_A_CSFWaterScaled	(Intercept)	Parametric	0	0.286	0	***	2.539
	tCr_A_CSFWaterScaled	Parametric	0.902	0.286	0.995		0.003
	IQ	Parametric	0.911	0.286	0.995		0
	eTIV	Parametric	0	0.286	0.001	***	0
	Sex - Female	Parametric	0.947	0.286	0.995		-0.002
	freqShift	Parametric	0.065	0.286	0.139		0.049
	s(age)	Smooth	0.001	0.286	0.002	**	1
Mean Voxel Cortical LGI - tCho_A_TissCorrWaterScaled	(Intercept)	Parametric	0	0.289	0	***	2.481
	tCho_A_TissCorrWaterScaled	Parametric	0.576	0.289	0.855		0.043
	IQ	Parametric	0.886	0.289	0.995		0
	eTIV	Parametric	0	0.289	0.001	***	0
	Sex - Female	Parametric	0.985	0.289	0.995		-0.001
	freqShift	Parametric	0.077	0.289	0.154		0.046
	s(age)	Smooth	0.001	0.289	0.002	**	1
Mean Voxel Cortical LGI - tCho_A_CSFWaterScaled	(Intercept)	Parametric	0	0.289	0	***	2.482
	tCho_A_CSFWaterScaled	Parametric	0.587	0.289	0.858		0.038
	IQ	Parametric	0.88	0.289	0.995		0
	eTIV	Parametric	0	0.289	0.001	***	0
	Sex - Female	Parametric	0.983	0.289	0.995		-0.001
	freqShift	Parametric	0.077	0.289	0.154		0.046
	s(age)	Smooth	0.001	0.289	0.002	**	1

Mean Voxel Cortical LGI - mI_A_TissCorrWaterScaled	(Intercept)	Parametric	0	0.296	0	***	2.391
	mI_A_TissCorrWaterScaled	Parametric	0.337	0.296	0.579		0.02
	IQ	Parametric	0.945	0.296	0.995		0
	eTIV	Parametric	0	0.296	0.001	***	0
	Sex - Female	Parametric	0.982	0.296	0.995		-0.001
	freqShift	Parametric	0.052	0.296	0.117		0.049
	s(age)	Smooth	0.001	0.296	0.002	**	1.149
Mean Voxel Cortical LGI - mI_A_CSFWaterScaled	(Intercept)	Parametric	0	0.296	0	***	2.392
	mI_A_CSFWaterScaled	Parametric	0.346	0.296	0.584		0.018
	IQ	Parametric	0.932	0.296	0.995		0
	eTIV	Parametric	0	0.296	0.001	***	0
	Sex - Female	Parametric	0.978	0.296	0.995		-0.001
	freqShift	Parametric	0.053	0.296	0.117		0.049
	s(age)	Smooth	0.001	0.296	0.002	**	1.148
Mean Voxel Cortical LGI - GlxGABAalpharatio (tissue-corrected)	(Intercept)	Parametric	0	0.288	0	***	2.631
	GlxGABAalpharatio	Parametric	0.688	0.288	0.964		-0.016
	IQ	Parametric	0.925	0.288	0.995		0
	eTIV	Parametric	0	0.288	0.001	***	0
	Sex - Female	Parametric	0.935	0.288	0.995		-0.003
	freqShift	Parametric	0.073	0.288	0.153		0.047
	s(age)	Smooth	0	0.288	0.001	**	1

CSF-corrected metabolites

A significant positive linear association was observed between PPC voxel mean cortical thickness and CSF-corrected GABA+ (beta = 0.06, p< 0.05) and CSF-corrected Glx (beta = 0.024, $p_{adjusted}$ < 0.05). CSF-corrected Glx also significantly positively associated with voxel cortical volume ($beta_{CSF\text{-}corrected}$ = 0.024, $p_{adjusted}$ < 0.05) and mean voxel LGI ($beta_{CSF\text{-}corrected}$ = 0.033, $p_{adjusted}$ < 0.05). CSF-corrected tCr significantly associated with voxel cortical area ($beta_{CSF\text{-}corrected}$ = -0.013, $p_{adjusted}$ < 0.05) and mean voxel cortical volume ($beta_{CSF\text{-}corrected}$ = -0.05, $p_{adjusted}$< 0.05).

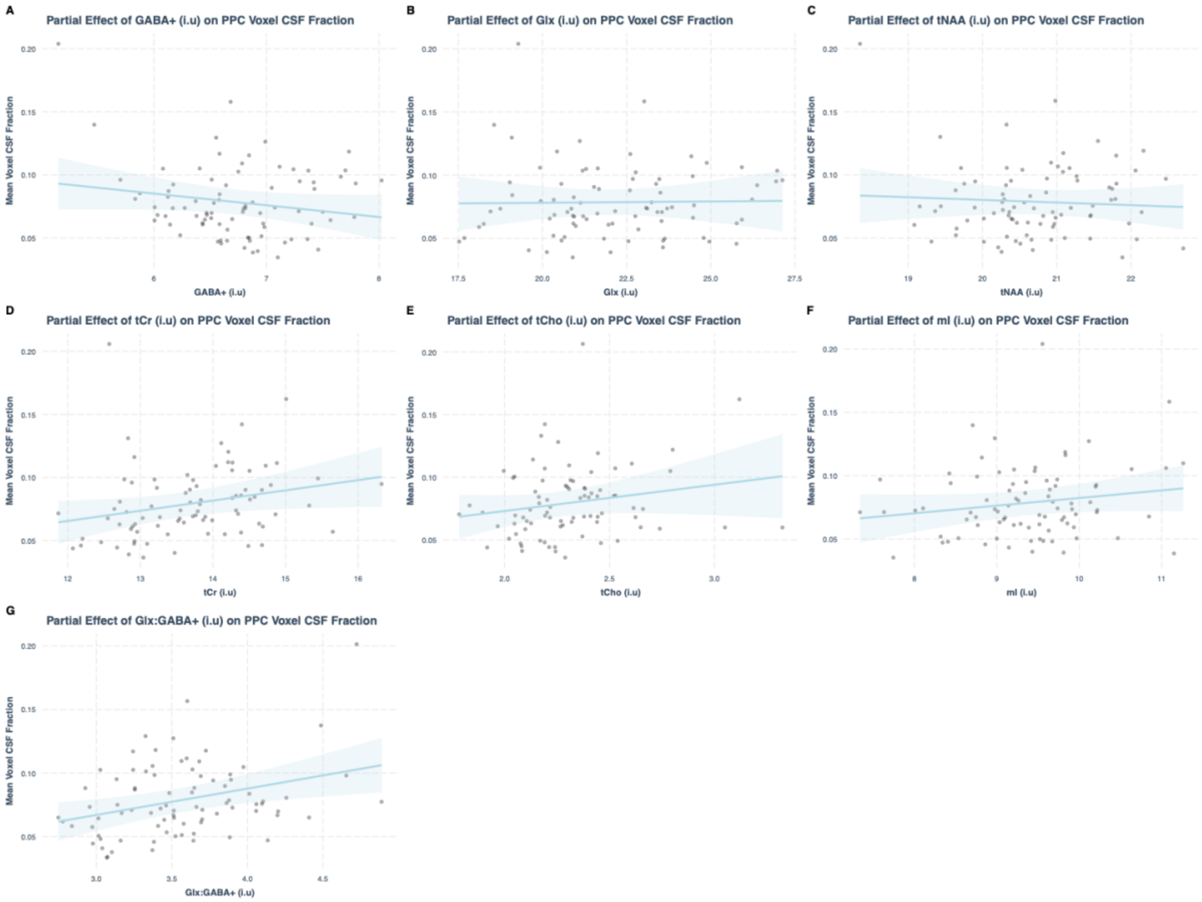

Supplementary Figure 5. Partial linear regression plot of PPC voxel CSF fraction predicted by tissue-corrected metabolite concentration holding participant age, sex, IQ, frequency shift and eTIV constant. Blue shading represents the 95% confidence interval for the partial regression prediction. Points represent individual partial residuals. A significant positive association between voxel CSF fraction and tissue-corrected Glx:GABA+ (beta = 0.021, $p_{adjusted} < 0.05$) was observed. No significant associations between metabolite levels and voxel GM or WM fraction were observed.
